# The 3C-like serine protease activity of porcine astrovirus nsP1a/3 mediates mitochondrial apoptosis and MAVS cleavage to facilitate viral replication and antagonize type I interferon response

**DOI:** 10.1371/journal.ppat.1013987

**Published:** 2026-02-17

**Authors:** YiYang Du, Yueqing Lv, Xiaoying Feng, Yuhang Luo, Xiaofang Wei, Sixiao Shao, Yeli Zhou, Kang Ouyang, Yeshi Yin, Ying Chen, Zuzhang Wei, Weijian Huang, Yifeng Qin

**Affiliations:** 1 Laboratory of Animal Infectious Diseases and Molecular Immunology, College of Animal Science and Technology, Guangxi University, Nanning, China; 2 Guangxi Zhuang Autonomous Region Engineering Research Center of Veterinary Biologics,; 3 Guangxi Key Laboratory of Animal Breeding, Disease Control and Prevention, Nanning, China; Universidad Nacional Autonoma de Mexico, MEXICO

## Abstract

Porcine astrovirus (PAstV) is globally prevalent in swine and is associated with diarrhea and encephalitis in piglets, posing a threat to porcine health. However, its pathogenic mechanisms remain poorly understood. This study used the PAstV1-GX1 strain to infect PK-15 cells, revealing that the virus induces significant apoptosis, with late apoptotic cells reaching 41.2% at 24 hours post-infection. The infection activates caspase-9 and caspase-3, but not caspase-8, and causes mitochondrial damage, indicating apoptosis via the mitochondrial pathway. The apoptosis inhibitor Z-VAD-FMK reduced viral replication, while apoptosis inducer ABT-263 enhanced it at later stages. The nsP1a/3 protein, which interacts with MAVS and localizes to mitochondria, was identified as key in inducing apoptosis. Its 3C-like serine protease domain likely mediates this interaction. Knocking down MAVS reduced apoptosis and increased early-stage replication but decreased it later. Overexpressing MAVS increased apoptosis and decreased replication. Furthermore, we observed that the expression of nsP1a/3 resulted in the cleavage of MAVS and the suppression of the type I interferon (IFN) response. Notably, treatment with Z-VAD-FMK did not influence nsP1a/3-mediated MAVS cleavage or type I IFN inhibition, suggesting that the induction of apoptosis and MAVS cleavage are distinct processes. By employing site-directed mutagenesis to substitute alanine for the catalytic triad residues (His_459_, Asp_487_, and Ser_549_) of the 3C-like serine protease, we significantly reduced the ability of nsP1a/3 to induce apoptosis, cleave MAVS, and suppress the type I IFN response, underscoring the essential role of protease activity in these functions. Furthermore, the use of a serine protease inhibitor markedly decreased PAstV replication. These findings provide significant insights into the pathogenesis of PAstV and establish a foundation for the development of novel antiviral therapies.

## Author summary

Porcine astrovirus (PAstV) is widespread in pigs, posing a threat to the pig industry’s sustainability, with no effective drugs or vaccines available. This study explored the mechanisms by which PAstV modulates apoptosis and innate immunity. Our findings indicate that PAstV infection triggers apoptosis through the mitochondrial pathway, which subsequently enhances viral replication during the later stages of infection. Additionally, we identified the viral protein nsP1a/3 as a critical factor that interacts with MAVS, localizes to mitochondria, and promotes apoptosis. Notably, our observations revealed that nsP1a/3 expression results in MAVS cleavage and a subsequent suppression of the type I IFN response. Importantly, the processes of nsP1a/3-induced apoptosis and MAVS cleavage were found to be independent. Site-directed mutagenesis of the catalytic triad (His_459_, Asp_487_, and Ser_549_) within the 3C-like serine protease domain of nsP1a/3 to alanine significantly reduced its capacity to induce apoptosis, cleave MAVS, and inhibit type I IFN production. Moreover, the application of a serine protease inhibitor significantly curtailed PAstV replication, highlighting the critical role of nsP1a/3 protease activity in both apoptosis induction and type I IFN suppression. These findings offer valuable insights into the pathogenesis of PAstV and establish a foundation for the development of innovative antiviral strategies.

## Introduction

Astroviruses (AstVs), members of the Astroviridae family, are characterized as non-enveloped, single-stranded, positive-sense RNA viruses [1]. The astrovirus genome, ranging from 6 to 8 kilobases, comprises three open reading frames (ORFs): ORF1a, ORF1b, and ORF2. ORF1a and ORF1b are situated at the 5’ end of the genome and encode nonstructural polyproteins nsP1a and nsP1ab. These polyproteins undergo proteolytic processing to yield RNA-dependent RNA polymerase, 3C-like serine protease, viral genome-linked protein (VPg), and several other proteins with as-yet-unknown functions [2]. ORF2, located at the 3’ end of the genome, encodes the viral capsid protein [3]. Based on host specificity, AstVs are classified into two genera: Mamastroviruses (MAstVs) and Avastroviruses (AAstVs) [4]. Porcine astrovirus (PAstV), a significant member of the MAstVs genus, was initially identified in piglet fecal samples in Canada in 1980 [5]. Currently, this virus is categorized into five genotypes (PAstV1-PAstV5), with notable regional differences in the prevalence of these genotypes [6]. Initial investigations indicated that PAstV was predominantly associated with intestinal infections. Nonetheless, recent research has demonstrated that PAstV is also capable of infecting the nervous and respiratory systems in pigs, resulting in conditions such as encephalitis and respiratory diseases [7–9]. This suggests that PAstV exhibits a broad tissue tropism and possesses complex pathogenic mechanisms.

Apoptosis, or programmed cell death, encompasses two primary pathways: the death receptor pathway (extrinsic apoptosis pathway) and the mitochondrial pathway (intrinsic apoptosis pathway). The death receptor pathway is facilitated by the Fas/FasL receptor and involves the activation of caspase-8. In contrast, the mitochondrial pathway is primarily initiated by the disruption of the mitochondrial membrane, leading to the release of cytochrome c and the activation of caspase-9. Both pathways ultimately converge on the activation of caspase-3 [10]. Apoptosis is crucial for maintaining intracellular homeostasis; however, it is also intricately linked to viral replication. Certain viruses facilitate their own replication by either inducing or inhibiting apoptosis. For instance, human astrovirus (HAstV) infection in Caco-2 cells enhances viral replication through the induction of apoptosis [11], while goose astrovirus (GAstV) infection in goslings leads to liver damage by triggering apoptosis in hepatic cells [12]. These findings underscore the significant association between apoptosis and both the replication and pathogenicity of AstVs. Nevertheless, the precise molecular mechanisms underlying apoptosis induction remain elusive, particularly as there is currently no published evidence elucidating the relationship between PAstV infection and apoptosis.

Innate immunity constitutes the primary defense mechanism against viral infections. Upon the entry of a virus into host cells, intracellular pattern recognition receptors (PRRs) identify viral nucleic acids, thereby initiating the type I interferon (IFN) signaling cascade. This activation results in the expression of numerous antiviral proteins, which collectively orchestrate a potent antiviral response [13]. Nevertheless, over the course of their evolution, many viruses have developed a variety of strategies to effectively suppress type I IFN production. A prevalent mechanism involves the targeting of mitochondrial antiviral signaling protein (MAVS). Certain viruses employ viral-encoded proteins to bind, cleave, or degrade MAVS, thereby disrupting the transduction of type I IFN signals [14,15]. Consequently, elucidating the molecular mechanisms by which viruses target MAVS to inhibit type I IFN responses is essential for advancing our understanding of viral pathogenesis.

In this study, we investigated the role of PAstV infection in apoptosis induction and elucidated the associated molecular pathways. Utilizing plasmid transfection methodologies, we identified a crucial viral protein responsible for apoptosis induction, demonstrating its initiation of this process through interaction with MAVS. Furthermore, the protein was found to suppress type I IFN production by cleaving MAVS. Notably, mutation of the 3C-like serine protease catalytic triad of this protein significantly inhibited both processes, and serine protease inhibitors were shown to markedly reduce PAstV replication. These findings provide substantial insights into the molecular mechanisms by which PAstV modulates apoptosis and innate immune signaling, thereby establishing a foundation for the development of antiviral strategies targeting PAstV.

## Result

### PAstV infection induces cell apoptosis

**Fig 1:**
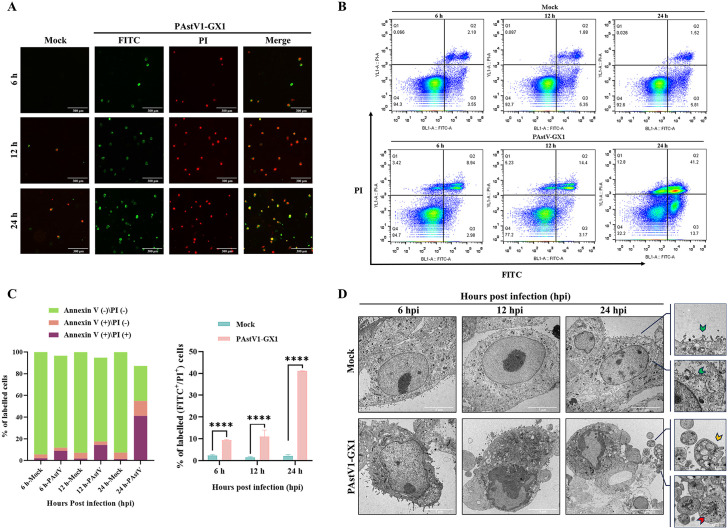
Apoptosis detection induced by PAstV infection. **(A, B)** PK-15 cells were infected with PAstV1-GX1 at an MOI of 0.1. The cells were subsequently stained using an Annexin V-FITC/PI Apoptosis Detection Kit, and apoptosis was assessed via fluorescence microscopy (A) and flow cytometry **(B)**. **(C)** The proportions of early apoptotic (Annexin V^+^/PI^-^) and late apoptotic (Annexin V^+^/PI^+^) cells identified through flow cytometry were quantified. Data are from three independent experiments (n = 3) and are presented as mean ± SD. Statistical analysis was performed using two-way ANOVA. Statistically significant differences compared to the mock-infected group are indicated by ****, p < 0.0001. **(D)** Transmission electron microscopy was employed to observe the morphological features of apoptotic cells induced by PAstV infection. The green arrow indicates intact cell membranes and organelles, while the yellow and red arrows denote apoptotic bodies containing damaged organelles and viral particles, respectively.

To examine the potential induction of apoptosis by PAstV infection, PK-15 cells were infected with the PAstV1-GX1 strain. Cell samples collected at 6, 12, and 24 hours post-infection (hpi) were stained using an Annexin V-FITC/PI Apoptosis Detection Kit and analyzed by fluorescence microscopy and flow cytometry. The results indicated that, in comparison to the mock-infected group, PAstV1-GX1 infection led to a significant increase in apoptotic cells at 12 and 24 hpi, as evidenced by an increased number of cells double-stained with Annexin V and PI (Fig 1A). Flow cytometry analysis demonstrated a progressive increase in the proportion of apoptotic cells with extended infection duration. By 24 hpi, the proportion of late apoptotic cells (Annexin V-FITC ⁺ /PI⁺) had reached 41.2% (Fig 1B). The PAstV1-GX1-infected group exhibited a significantly higher proportion of apoptotic cells compared to the mock-infected group at all assessed time points (p < 0.0001) (Fig 1C). Concurrently, transmission electron microscopy (TEM) of PAstV-infected PK-15 cells revealed distinct apoptotic morphological features at 12 and 24 hpi, including nuclear condensation, chromatin margination, and a notable increase in cytoplasmic vesicles. By 24 hpi, apoptotic bodies containing damaged organelles and electron-dense viral particles were observed detaching from the cell membrane, whereas the mock-infected group did not display any discernible apoptotic morphology (Fig 1D).

### PAstV induces apoptosis via the mitochondrial pathway

**Fig 2:**
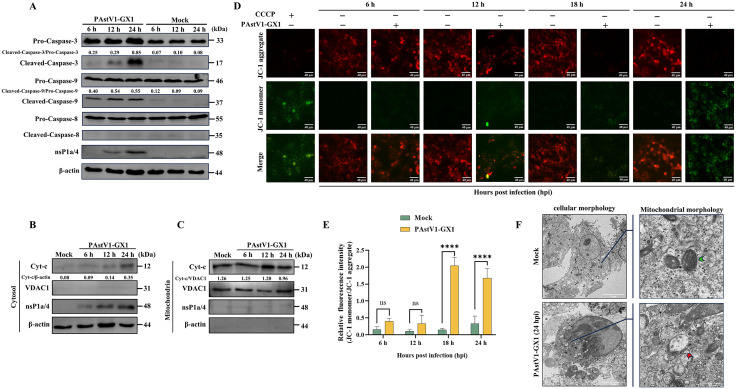
PAstV infection causes mitochondrial damage and induces apoptosis through the mitochondrial pathway. **(A)** PK-15 cells were either mock-infected or infected with PAstV1-GX1 (MOI = 0.1). Cell lysates were collected at 6, 12, and 24 hpi and then subjected to Western blot analysis using antibodies specific for caspase-3, caspase-9, caspase-8, PAstV nsP1a/4, and β-actin. The relative expression ratios of cleaved caspase-3 to pro-caspase-3 and cleaved caspase-9 to pro-caspase-9 were quantified by grayscale analysis using ImageJ software. **(B, C)** PK-15 cells infected with PAstV1-GX1 were subjected to subcellular fractionation to isolate cytoplasmic and mitochondrial fractions. The cytochrome c expression levels in the cytoplasmic (B) and mitochondrial (C) fractions were detected by Western blot analysis; β-actin and VDAC1 served as loading controls for the cytoplasmic and mitochondrial fractions, respectively. **(D, E)** Mitochondrial membrane potential (ΔΨm) was assessed in PAstV1-GX1-infected and mock-infected cells by using the JC-1 probe, with CCCP treatment (10 µM) serving as a positive control **(D)**. The relative fluorescence intensity ratio of green to red fluorescence (JC-1 monomer to JC-1 aggregate) was quantified using ImageJ software **(E)**. Bar graphs display the mean ± SD from three independent experiments (n = 3). Statistical analysis was performed using two-way ANOVA, with significant differences versus the mock-infected group are indicated by ****, p < 0.0001. **(F)** Representative transmission electron microscopy (TEM) images showing cellular and mitochondrial morphology in cells of the PAstV1-GX1-infected and mock-infected groups. The green and red arrows denote normal and damaged mitochondria, respectively.

Apoptosis is predominantly initiated via two distinct pathways: the death receptor pathway and the mitochondrial pathway. To elucidate the primary pathway implicated in PAstV-induced apoptosis, PK-15 cells were infected with the PAstV1-GX1 strain, and the activation status of caspase-3, caspase-8, and caspase-9 was evaluated using Western blot analysis. The findings revealed that PAstV infection significantly induced the activation of caspase-3 and caspase-9, with the expression levels of cleaved caspase-3 and caspase-9 progressively increasing over time. In contrast, no cleavage of caspase-8 was detected in infected cells. Furthermore, mock-infected controls showed no activation of caspase-3, -8, or -9 (Fig 2A). These results suggest that PAstV infection predominantly induces apoptosis via the mitochondrial pathway. To examine the impact of PAstV infection on mitochondrial integrity, PK-15 cells infected with PAstV1-GX1 were subjected to fractionation into cytoplasmic and mitochondrial components at various post-infection time points. Western blot analysis of cytochrome c expression indicated a progressive increase in cytoplasmic cytochrome c levels (Fig 2B), accompanied by a corresponding decrease in mitochondrial fractions as the infection advanced (Fig 2C). These findings suggest that PAstV infection facilitates the translocation of cytochrome c from mitochondria to the cytosol. Corroborating these results, assessments of mitochondrial membrane potential (ΔΨm) demonstrated a gradual decline in ΔΨm levels throughout the course of PAstV1-GX1 infection, evidenced by a progressive reduction in red fluorescence intensity and a concomitant increase in green fluorescence (Fig 2D and 2E). Moreover, ultrastructural analysis by electron microscopy revealed that PAstV1-GX1 infection induced substantial mitochondrial damage, characterized by swelling, cristae disruption, and vacuolization, whereas mitochondria in uninfected control cells maintained normal morphology (Fig 2F). Collectively, these findings demonstrate that PAstV infection compromises mitochondrial integrity, resulting in cytochrome c release and subsequent activation of the caspase-9/-3 cascade, thereby predominantly inducing apoptosis via the mitochondrial pathway.

### Apoptosis promotes PAstV replication

**Fig 3:**
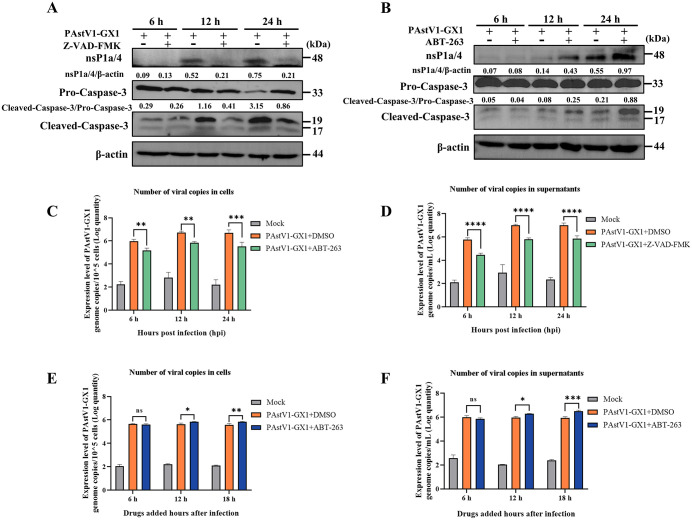
Analysis of apoptosis modulation on PAstV replication. **(A, B)** PK-15 cells were infected with PAstV1-GX1 (MOI = 0.1) and treated with or without Z-VAD-FMK (10 μM) (A) or ABT-263 (5 μM) **(B)**. Cell lysates were collected at 6, 12, and 24 hpi and subjected to Western blot analysis using antibodies against caspase-3, PAstV nsP1a/4, and β-actin. The relative expression ratios of nsP1a/4 to β-actin and cleaved caspase-3 to pro-caspase-3 were quantified by grayscale analysis using ImageJ software. **(C, D)** PK-15 cells were infected with PAstV1-GX1 (MOI = 0.1) and treated with or without Z-VAD-FMK (10 μM). Intracellular (C) and extracellular (D) viral RNA levels at the indicated time points post-infection were quantified by RT-qPCR. **(E, F)** PK-15 cells infected with PAstV1-GX1 (MOI = 0.1) were treated with ABT-263 (5 μM) at 6, 12, or 18 hpi. Intracellular (E) and extracellular (F) viral RNA copies were measured at 24 hpi using RT-qPCR. Data are presented as mean ± SD from three independent experiments (n = 3). Statistical analyses were conducted using two-way ANOVA. Statistical significance compared to the untreated control is indicated as *p < 0.05, **p < 0.01, ***p < 0.001, and ****p < 0.0001.

To examine the impact of apoptosis on PAstV replication, PK-15 cells were infected with the PAstV1-GX1 strain and subsequently treated with either the apoptosis inhibitor Z-VAD-FMK or the apoptosis inducer ABT-263. The expression levels of the PAstV nsP1a/4 protein were analyzed via Western blotting at various time points post-infection. The results showed that, relative to the untreated control group, treatment with Z-VAD-FMK inhibited caspase-3 cleavage and significantly reduced the expression of the nsP1a/4 protein at 12 and 24 hpi (Fig 3A). In contrast, ABT-263 treatment facilitated caspase-3 cleavage and significantly increased nsP1a/4 protein expression at these same time points (Fig 3B). In addition, cell and supernatant samples were collected at 6, 12, and 24 hpi, and the copy numbers of PAstV mRNA were quantified using RT-qPCR. Treatment with Z-VAD-FMK resulted in a significant reduction in viral copy numbers in both cells and supernatants compared to the untreated group (p < 0.01) (Fig 3C and 3D). Conversely, treatment with ABT-263 concurrent with infection did not result in a significant alteration in viral copy numbers at any time point when compared to the untreated group (p > 0.05) (S1 Fig). To further assess the impact of ABT-263-induced apoptosis on PAstV replication, ABT-263 was administered at 6, 12, or 18 hpi, with subsequent collection of cell and supernatant samples at 24 hpi for RT-qPCR analysis. Relative to the untreated control, treatment at 6 hpi did not significantly affect viral copy numbers. However, treatment at 12 or 18 hpi significantly increased viral copy numbers in both cells and supernatants compared to the untreated control (p < 0.05) (Fig 3E and 3F). Collectively, these findings suggest that the inhibition of apoptosis via Z-VAD-FMK suppresses PAstV replication, whereas the induction of apoptosis through ABT-263 enhances PAstV replication.

### The nsP1a/3 is the key PAstV protein triggering mitochondria-mediated apoptosis

**Fig 4:**
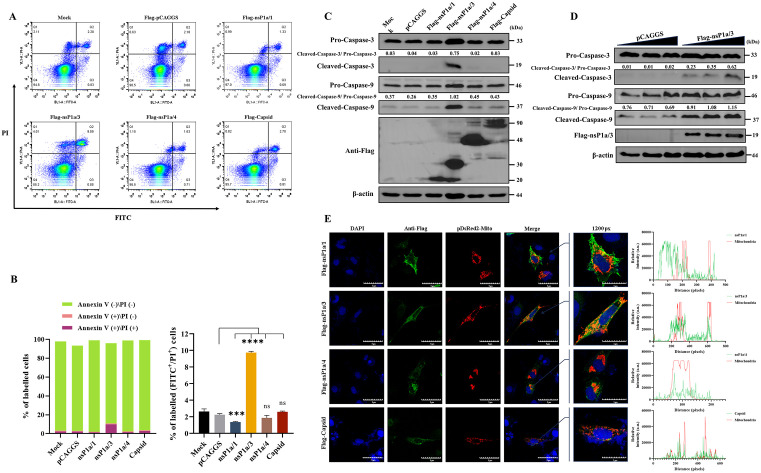
Identification of key viral proteins involved in PAstV-induced apoptosis. **(A, B)** PK-15 cells were transfected with eukaryotic expression plasmids encoding nsP1a/1, nsP1a/3, nsP1a/4, capsid protein, or an empty vector control. Apoptosis was assessed at 24 hours post-transfection by Annexin V-FITC/PI staining and flow cytometry. Representative flow cytometry plots are shown in **(A)**. The proportions of early apoptotic (Annexin V ⁺ /PI⁻) and late apoptotic (Annexin V ⁺ /PI⁺) cells were quantified in **(B)**. Data are from three independent experiments (n = 3) and are presented as mean ± SD. Statistical analyses were conducted using one-way ANOVA. Statistically significant differences compared to the empty vector group are indicated as **p < 0.01 and ****p < 0.0001. **(C)** PK-15 cells were transfected with the indicated plasmids. Cell lysates were collected at 24 hours post-transfection and subjected to Western blot analysis using antibodies specific for caspase-3, caspase-9, and β-actin. The relative expression ratios of cleaved caspase-3 to pro-caspase-3 and cleaved caspase-9 to pro-caspase-9 were quantified by grayscale analysis using ImageJ software. **(D)** PK-15 cells were transfected with increasing amounts (1, 2.5, or 5 µg) of pCAGGS-Flag-nsP1a/3 or empty vector for 24 hours. Western blot analysis was performed on cell lysates using antibodies against caspase-3, caspase-9, Flag, and β-actin. The relative expression ratios of cleaved caspase-3 to pro-caspase-3 and cleaved caspase-9 to pro-caspase-9 were quantified by grayscale analysis using ImageJ software. **(E)** BHK-21 cells were subjected to co-transfection with pDsRed2-Mito and the specified viral protein expression plasmids. At 24 hours post-transfection, confocal microscopy was utilized to evaluate the co-localization of PAstV proteins (green), tagged with an anti-Flag antibody and a CoraLite Plus 488-conjugated secondary antibody, with mitochondria (red), marked by pDsRed2-Mito. The fluorescence intensity profiles of viral proteins (green) and mitochondria (red) along a defined line were quantified using ImageJ. Scale bar, 5 μm.

To elucidate the key proteins of PAstV implicated in apoptosis induction, eukaryotic expression plasmids encoding PAstV nsP1a/1, nsP1a/3, nsP1a/4, and the capsid protein, alongside an empty vector control plasmid (pCAGGS), were transfected into PK-15 cells. Apoptosis was evaluated 24 hours post-transfection utilizing an Annexin V-FITC/PI Apoptosis Detection Kit in conjunction with flow cytometry. The findings revealed that the expression of nsP1a/3, nsP1a/4, and the capsid protein significantly increased the proportion of apoptotic cells compared to the empty vector control group (p < 0.01), with nsP1a/3 expression causing the most substantial apoptosis. Notably, the proportion of late apoptotic cells (Annexin V-FITC + /PI+) induced by nsP1a/3 expression reached approximately 10.3%. In contrast, the apoptotic cell proportion resulting from nsP1a/1 expression did not significantly differ from that of the control group (p > 0.05) (Fig 4A and 4B). Western blot analysis of total cellular proteins harvested 24 hours post-transfection demonstrated that only nsP1a/3 expression led to significant cleavage of caspase-3 and caspase-9, whereas the expression of nsP1a/1, nsP1a/4, and the capsid protein did not result in notable cleavage of these caspases (Fig 4C). To further validate these observations, PK-15 cells were transfected with progressively increasing concentrations of the nsP1a/3 eukaryotic expression plasmid. The results demonstrated a dose-dependent augmentation in the cleavage of caspase-3 and caspase-9, which was concomitant with elevated expression levels of nsP1a/3 (Fig 4D). Furthermore, to explore potential mitochondrial involvement, BHK-21 cells were co-transfected with eukaryotic expression plasmids encoding nsP1a/1, nsP1a/3, nsP1a/4, or the capsid protein, in conjunction with the mitochondrial marker plasmid pDsRed2-Mito. Analysis via laser scanning confocal microscopy revealed that only the nsP1a/3 protein exhibited significant co-localization with cellular mitochondria, whereas the other viral proteins did not (Fig 4E). Collectively, these findings suggest that the nsP1a/3 protein is the primary viral protein implicated in PAstV-induced apoptosis through the mitochondria-mediated pathway, with its expression specifically localizing to the mitochondria.

### PAstV nsP1a/3 directly interacts with mitochondrial antiviral signaling protein MAVS

**Fig 5:**
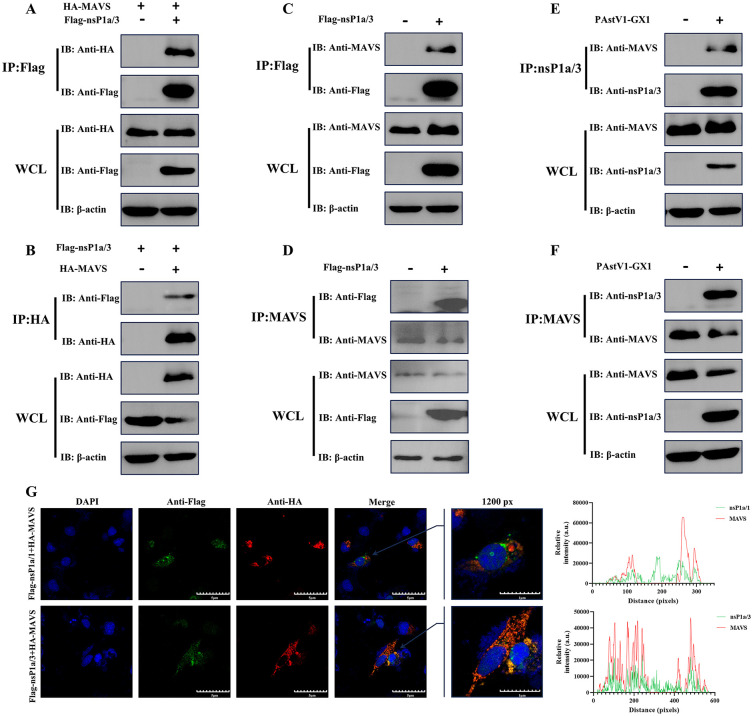
Identification of nsP1a/3 protein interaction with MAVS. **(A, B)** HEK-293T cells were co-transfected with pCAGGS-Flag-nsP1a/3 and pCAGGS-HA-MAVS plasmids for 24 hours. Cell lysates were subjected to co-IP with anti-Flag (A) or anti-HA beads **(B)**. The precipitated proteins, along with whole-cell lysates (WCL), were analyzed by Western blot using anti-HA and anti-Flag antibodies. **(C, D)** PK-15 cells were transfected with plasmid pCAGGS-Flag-nsP1a/3 for 24 hours. Lysates were subjected to co-IP with anti-Flag (C) or anti-MAVS (D) beads. The precipitated proteins and WCL were then analyzed by Western blot using anti-MAVS and anti-Flag antibodies. **(E, F)** PK-15 cells were infected with PAstV1-GX1 at an MOI of 0.1 for 24 hours. The lysates were subjected to co-IP with anti-nsP1a/3 (E) or anti-MAVS (F) beads. The precipitated proteins and WCL were analyzed by Western blot using anti-MAVS and anti-nsP1a/3 antibodies. β-actin was used as a loading control. **(G)** BHK-21 cells were co-transfected with pCAGGS-HA-MAVS and either pCAGGS-Flag-nsP1a/3 or pCAGGS-Flag-nsP1a/1. After 24 hours, confocal immunofluorescence was conducted using anti-Flag and anti-HA antibodies, with nuclei stained by DAPI. The right panels display fluorescence intensity profiles of Flag-tagged nsP1a/1 or nsP1a/3 (green) and HA-tagged MAVS (red) measured using ImageJ. Scale bar: 5 μm.

As described above, we observed the co-localization of the PAstV nsP1a/3 protein with cellular mitochondria. To identify mitochondrial proteins interacting with nsP1a/3, HEK-293T cells were co-transfected with a Flag-tagged nsP1a/3 expression plasmid and HA-tagged expression plasmids encoding mitochondrial antiviral signaling protein (MAVS) or voltage-dependent anion channels 1 and 2 (VDAC1 and VDAC2). Protein interactions were assessed through co-immunoprecipitation (Co-IP) 24 hours post-transfection. Reciprocal Co-IP assays confirmed a specific interaction between nsP1a/3 and MAVS: Immunoprecipitation of the Flag-tagged nsP1a/3 resulted in the co-precipitation of HA-tagged MAVS, while reciprocal immunoprecipitation of HA-tagged MAVS successfully co-precipitated Flag-tagged nsP1a/3 (Fig 5A and 5B). Notably, no interaction was observed between Flag-nsP1a/3 and HA-tagged VDAC1 or VDAC2 in either assay configuration (S2 Fig). To confirm physiological relevance, reciprocal Co-IP was conducted in porcine PK-15 cells transfected with the Flag-nsP1a/3 plasmid (Fig 5C and 5D) or infected with PAstV1-GX1 strain (Fig 5E and 5F). Both the overexpressed nsP1a/3 and the viral infection-derived nsP1a/3 demonstrated interaction with endogenous MAVS, thereby affirming the interaction under physiologically relevant conditions. These findings were further validated through laser scanning confocal microscopy, which demonstrated significant intracellular co-localization between nsP1a/3 and MAVS. In contrast, the control protein nsP1a/1 did not exhibit co-localization with MAVS (Fig 5G), highlighting the specificity of the nsP1a/3–MAVS interaction. To explore this interaction in greater detail, the full-length sequences of both proteins were analyzed using AlphaFold 3 for 3D structural prediction. Subsequent Rosetta docking analysis produced a complex model with a docking free energy of –43.94 kcal/mol, a docking score of –284.81, and an overall confidence score of 0.9368, indicating a high level of model reliability. The model reveals a substantial binding interface between the 3C-like serine protease domain of nsP1a/3 and MAVS, supported by 10 hydrogen bonds and one hydrophobic interaction (S1 Table and S3 Fig), suggesting a strong binding affinity. These results suggest that the 3C-like serine protease domain within nsP1a/3 may act as a critical region mediating its interaction with MAVS. In our previous research, we identified that the PAstV nsP1a/4 protein is capable of interacting with human MAVS [16]. Consequently, in this study, we further investigated the interaction between nsP1a/4 and porcine MAVS, discovering that the nsP1a/4 protein also interacts with porcine MAVS (S4 Fig).

### MAVS knockdown suppresses PAstV-induced apoptosis but exerts biphasic regulation on viral replication: early promotion and late suppression

**Fig 6:**
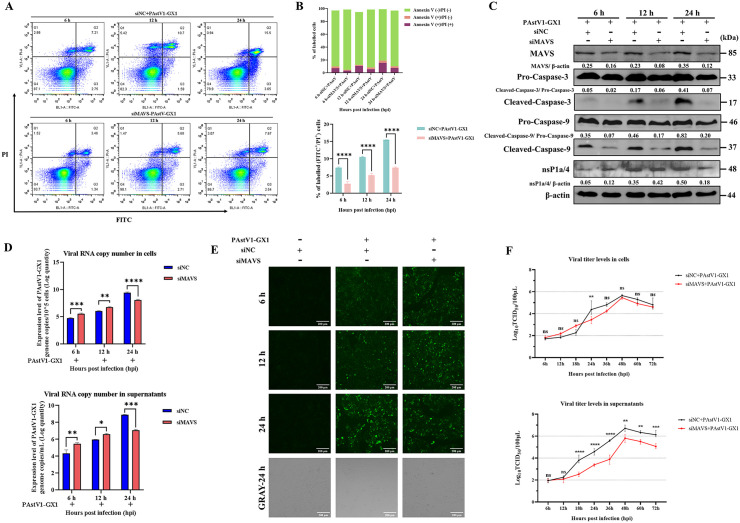
Effect of MAVS knockdown on PAstV1-GX1-induced apoptosis and viral replication in PK-15 cells. **(A, B)** PK-15 cells were transfected with MAVS-targeting siRNA (siMAVS) or control siRNA (siNC) and then infected with PAstV1-GX1 (MOI = 0.1). Apoptosis was assessed at 6, 12, and 24 hpi using Annexin V-FITC/PI staining and flow cytometry. Representative flow cytometry plots are shown in (A). The proportions of early apoptotic (Annexin V ⁺ /PI⁻) and late apoptotic (Annexin V ⁺ /PI⁺) cells were quantified in **(B).** Data are from three independent experiments (n = 3) and are presented as mean ± SD. Statistical analysis was performed using two-way ANOVA. Statistically significant differences compared to the siNC group are indicated as ****, p < 0.0001. **(C)** PK-15 cells transfected with siMAVS or siNC were infected with PAstV1-GX1 (MOI = 0.1). Cell lysates were harvested at 24 hpi and subjected to Western blot analysis using antibodies against MAVS, caspase-3, caspase-9, nsP1a/4, and β-actin. The relative expression ratios of MAVS to β-actin, cleaved caspase-3 to pro-caspase-3, cleaved caspase-9 to pro-caspase-9, and nsP1a/4 to β-actin were quantified using ImageJ software for grayscale analysis. **(D)** Viral RNA levels in the cell lysates (upper panel) and supernatants (lower panel) of cells transfected with siMAVS or siNC were measured by RT-qPCR at 6, 12, and 24 hpi. Data are presented as mean ± SD from three experiments (n = 3). Statistical analysis was performed using two-way ANOVA, with significant differences from the siNC group noted as *p < 0.05, **p < 0.01, ***p < 0.001, ****p < 0.0001. **(E)** PK-15 cells transfected with siMAVS or siNC were infected with PAstV1-GX1 (MOI = 1). At 6, 12, and 24 hpi, cells were processed for IFA using a mouse anti-nsP1a/4 primary antibody and an FITC-conjugated goat anti-mouse IgG secondary antibody, and then observed by fluorescence microscopy. **(F)** Viral growth kinetics were determined by TCID₅₀ assay using cell and supernatant samples collected from siMAVS- or siNC-transfected PK-15 cells at the indicated times after infection with PAstV1-GX1 (MOI = 0.1). Data are presented as mean ± SD from three independent experiments (n = 3). Statistical analysis was performed using two-way ANOVA. Statistically significant differences compared to the siNC group are indicated as ** p < 0.01, ***p < 0.001, ****p < 0.0001.

To elucidate the role of MAVS in PAstV-induced apoptosis and viral replication, PK-15 cells were transfected with MAVS-specific siRNA (siMAVS) or control siRNA (siNC) and subsequently infected with the PAstV1-GX1 strain. Apoptosis was evaluated using flow cytometry with Annexin V-FITC/PI at 6, 12, and 24 hpi. The results demonstrated that MAVS-knockdown (MAVS-KD) cells exhibited a significantly lower proportion of apoptotic cells at all assessed time points post-infection compared to siNC-treated cells (p < 0.0001; refer to Fig 6A and 6B). Supporting these observations, Western blot analysis revealed a marked reduction in the expression levels of cleaved caspase-3 and cleaved caspase-9 in MAVS-KD cells relative to siNC-treated cells following infection (Fig 6C), indicating that MAVS knockdown effectively suppresses PAstV-induced apoptosis. Notably, the expression of the PAstV nsP1a/4 protein in MAVS-KD cells was significantly upregulated at 6 and 12 hpi but significantly downregulated at 24 hpi compared to siNC-treated cells (Fig 6C). Additionally, CCK-8 assays indicated that transfection of PK-15 cells with 0.5, 1.0, 2.5, or 5 µg of siMAVS did not result in a significant impact on cell viability (S5 Fig).

To further evaluate the effect of MAVS knockdown on PAstV replication, viral copy numbers in both cells and supernatants were quantified using RT-qPCR at 6, 12, and 24 hpi. In alignment with the nsP1a/4 protein expression findings, viral copy numbers were significantly elevated in both cells and supernatants of MAVS-KD cells compared to those treated with siNC at 6 and 12 hpi (*p* < 0.05). Conversely, at 24 hpi, viral copy numbers were markedly reduced in MAVS-KD cells (*p* < 0.001) (Fig 6D). Immunofluorescence assay (IFA) further corroborated the enhanced viral replication in MAVS-KD cells at 6 and 12 hpi, as indicated by increased fluorescence intensity specific to PAstV antigens relative to siNC-treated cells. In contrast, greater fluorescence intensity was observed in siNC-treated cells at 24 hpi (Figs 6E and S6). The virus multi-step growth curve analysis further elucidated this biphasic effect. Although intracellular virus titers in MAVS-KD cells were consistently higher than those in siNC-treated cells between 6 and 18 hpi, the difference did not achieve statistical significance (*p* > 0.05). However, from 24 to 72 hpi, intracellular titers were significantly lower in MAVS-KD cells, with a pronounced reduction observed at 24 hpi (*p* < 0.01; refer to Fig 6F). Additionally, supernatant virus titers in MAVS-KD cells were significantly lower than those in siNC-treated cells from 18 to 72 hpi (p < 0.01; refer to Fig 6F). Collectively, these findings indicate that MAVS knockdown inhibits PAstV-induced apoptosis and exerts a biphasic effect on viral replication. Specifically, it enhances PAstV replication during the early infection phase (6–18 hpi) while suppressing it in the later stages (24–72 hpi). This suppression in the later stages may be attributed to impeded viral release or possibly impaired capsid cleavage and virus maturation.

### MAVS overexpression enhances PAstV-induced apoptosis while suppressing viral replication

**Fig 7:**
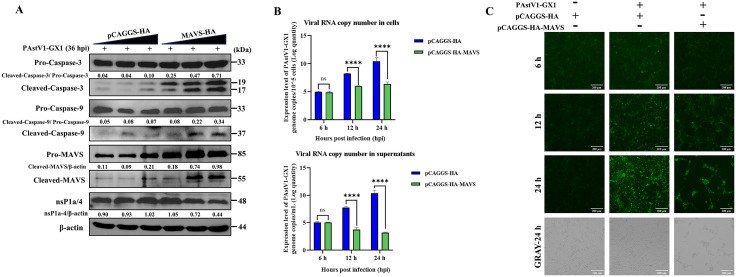
Effect of MAVS overexpression on PAstV1-GX1-induced apoptosis and viral replication in PK-15 cells. **(A)** PK-15 cells were transfected with increasing amounts (1, 2.5, and 5 μg) of pCAGGS-HA-MAVS or empty vector prior to infection with PAstV1-GX1 (MOI = 0.1). At 24 hpi, cell lysates were analyzed by Western blot using antibodies against caspase-3, caspase-9, MAVS, nsP1a/4, and β-actin. The relative expression ratios of cleaved caspase-3 to pro-caspase-3, cleaved caspase-9 to pro-caspase-9, cleaved MAVS to β-actin, and nsP1a/4 to β-actin were quantified using ImageJ software for grayscale analysis. **(B)** RT-qPCR quantification of PAstV1-GX1 viral RNA levels in cell lysates (upper panel) and supernatants (lower panel) from PK-15 cells transfected with pCAGGS-HA-MAVS or empty vector. Data represent mean ± SD from three independent experiments (n = 3). Statistical analysis was performed using two-way ANOVA, with statistically significant differences compared to the empty vector control group denoted by ****p < 0.0001. **(C)** PK-15 cells transfected with pCAGGS-HA-MAVS or empty vector were infected with PAstV1-GX1 (MOI = 1). At 6, 12, and 24 hpi, cells were processed for IFA using a mouse anti-nsP1a/4 primary antibody and an FITC-conjugated goat anti-mouse IgG secondary antibody, followed by observation under a fluorescence microscope.

In the aforementioned study, it was observed that the knockout of MAVS inhibited PAstV-induced apoptosis in cells, facilitated early viral replication, but subsequently impeded it. To further examine the impact of MAVS overexpression on PAstV-induced apoptosis and replication, PK-15 cells were transfected with varying concentrations of a eukaryotic MAVS expression plasmid or an empty vector control (pCAGGS). Total cellular proteins were collected 24 hours post-transfection for analysis via Western blotting. The results demonstrated that increased MAVS expression resulted in elevated levels of cleaved caspase-3 and cleaved caspase-9 following PAstV1-GX1 infection, while the expression of the PAstV nsP1a/4 protein progressively declined (Fig 7A). These findings suggest that overexpression of MAVS enhances PAstV-induced apoptosis and concurrently suppresses PAstV replication. Importantly, infection with PAstV1-GX1 led to the cleavage of MAVS, resulting in the generation of an additional fragment approximately 55 kDa in size, with its abundance increasing in proportion to MAVS overexpression (Fig 7A). To further evaluate PAstV replication, PK-15 cells were transfected and subsequently infected with PAstV1-GX1, RT-qPCR analysis demonstrated a significant reduction in PAstV RNA copy numbers in both the cells and supernatants of the MAVS-overexpression group compared to the control group at 12 and 24 hpi (p < 0.0001) (Fig 7B). Correspondingly, immunofluorescence assays revealed diminished fluorescence intensity in the MAVS-overexpression group relative to the control group at 6, 12, and 24 hpi. Notably, cells overexpressing MAVS exhibited more pronounced cytopathic effects (CPE), characterized by increased cell rounding, detachment, and death (Fig 7C). In summary, these findings demonstrate that MAVS overexpression enhances PAstV-induced apoptosis while inhibiting viral replication.

### PAstV nsP1a/3 protein cleaves MAVS and antagonizes type I interferon production

**Fig 8:**
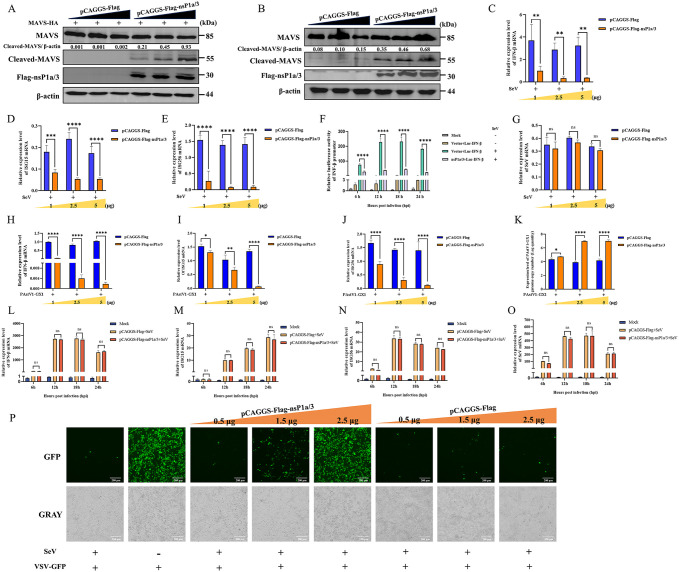
PAstV nsP1a/3 cleaves MAVS and inhibits type I IFN production. **(A)** PK-15 cells were co-transfected with 1 µg of pCAGGS-HA-MAVS and increasing amounts of pCAGGS-Flag-nsP1a/3 or empty vector control. Cell lysates were harvested 24 h post-transfection and analyzed by Western blot using antibodies against HA, Flag, and β-actin. The relative ratio of cleaved exogenous MAVS to β-actin was quantified via grayscale analysis using ImageJ software. **(B)** PK-15 cells were transfected with increasing amounts of pCAGGS-Flag-nsP1a/3 or empty vector control. Cell lysates were collected at 24 h post-transfection and subjected to Western blot analysis using antibodies targeting MAVS, Flag, and β-actin. The relative expression level of cleaved endogenous MAVS to β-actin was quantified by grayscale analysis with ImageJ software. (C, D, **E)** PK-15 cells were transfected with increasing amounts of pCAGGS-Flag-nsP1a/3 or empty vector control, followed by infection with SeV (10 HAU). The relative mRNA levels of porcine IFN-β **(C)**, ISG15 **(D)**, and ISG56 (E) were assessed by RT-qPCR and normalized to porcine β-actin. **(F)** PK-15 cells were co-transfected with pCAGGS-Flag-nsP1a/3 or empty vector control, along with pIFN-β-Luc and pRL-TK reporter plasmids, and then infected with SeV. Luciferase activities were measured at 6, 12, and 24 hpi. **(G)** PK-15 cells were transfected with increasing amounts of pCAGGS-Flag-nsP1a/3 or empty vector control, followed by infection with SeV (10 HAU). The relative mRNA levels of SeV were assessed by RT-qPCR and normalized to porcine β-actin. (H, I, **J)** PK-15 cells were transfected with increasing amounts of pCAGGS-Flag-nsP1a/3 or empty vector control, followed by infection with PAstV1-GX1 at an MOI of 3. The relative mRNA levels of porcine IFN-β **(H)**, ISG15 **(I)**, and ISG56 (J) were assessed by RT-qPCR and normalized to porcine β-actin. **(K)** PK-15 cells were transfected with increasing amounts of pCAGGS-Flag-nsP1a/3 or empty vector control and subsequently infected with PAstV1-GX1 at an MOI of 3. PAstV mRNA copy numbers were measured by RT-qPCR at 24 hpi. (L, M, N, **O)** HEK 293T cells were transfected with pCAGGS-Flag-nsP1a/3 or empty vector control, followed by infection with SeV (10 HAU). The mRNA expression levels of human IFN-β **(L)**, ISG15 **(M)**, ISG56 **(N)**, and SeV (O) were determined by RT-qPCR and normalized to human β-actin. **(P)** PK-15 cells were transfected with increasing amounts of pCAGGS-Flag-nsP1a/3 or empty vector control. At 24 hours post-transfection, the cells were infected with SeV for 12 hours, and the culture supernatants were collected. Following UV irradiation, the supernatants were transferred to fresh PK-15 cells. After 24 hours of incubation, these recipient cells were infected with VSV-GFP. Viral replication was assessed 12 hours later by fluorescence microscopy. Data are presented as mean ± SD from three independent experiments (n = 3). Statistical analysis was performed using two-way ANOVA. Significant differences compared to the empty vector control group are indicated as *p < 0.05, **p < 0.01, ***p < 0.001, and ****p < 0.0001.

In the aforementioned study, it was observed that infection with PAstV1-GX1 leads to the cleavage of MAVS. Given the interaction between the nsP1a/3 protein and MAVS, we hypothesized that nsP1a/3 is responsible for facilitating this cleavage. To test this hypothesis, PK-15 cells were co-transfected with pCAGGS-HA-MAVS and varying concentrations (1, 2.5, or 5 µg) of either pCAGGS-Flag-nsP1a/3 or the control vector pCAGGS. Western blot analysis revealed a dose-dependent increase in the levels of cleaved exogenous MAVS, which corresponded with elevated expression of nsP1a/3 (Fig 8A). To validate these results and examine the cleavage of endogenous MAVS, PK-15 cells were transfected with escalating amounts (1, 2.5, or 5 µg) of the nsP1a/3 plasmid or the control vector pCAGGS. Subsequent Western blotting, performed 24 hours post-transfection, demonstrated a dose-dependent increase in cleaved endogenous MAVS levels (Fig 8B), implying that the PAstV nsP1a/3 protein specifically targets and cleaves MAVS. Additionally, to assess whether the interaction between nsP1a/4 and MAVS also results in MAVS cleavage, PK-15 cells were transfected with escalating amounts (1, 2.5, or 5 µg) of the nsP1a/4 plasmid. Western blot analysis indicated that nsP1a/4 expression did not induce cleavage of endogenous MAVS (S7 Fig).

Given the pivotal role of MAVS as an adaptor in type I IFN signaling, we explored the potential antagonistic effects of nsP1a/3-mediated MAVS cleavage on type I IFN responses. To this end, PK-15 cells were transfected with increasing amounts of nsP1a/3 plasmid or a pCAGGS vector and subsequently infected with Sendai virus (SeV). Quantitative RT-PCR analysis revealed that nsP1a/3 overexpression led to a dose-dependent and statistically significant suppression of SeV-induced mRNA expression of IFN-β (Fig 8C), ISG15 (Fig 8D), and ISG56 (Fig 8E) when compared to the vector control (*p* < 0.01). Furthermore, dual-luciferase reporter assays indicated that SeV infection significantly activated IFN-β promoter activity at 6, 12, 18, and 24 hpi. Importantly, nsP1a/3 overexpression markedly reduced this promoter activity at all examined time points (p < 0.0001; Fig 8F). Notably, RT-qPCR quantification of SeV HN gene copies demonstrated no significant difference in viral replication between cells overexpressing nsP1a/3 and those with the vector control (p > 0.05; Fig 8G), suggesting that nsP1a/3 does not directly impede SeV replication. Given that PAstV1-GX1 infection itself induces type I IFN production [17], we next examined whether nsP1a/3 antagonizes type I IFN induction during authentic PAstV infection. In alignment with the results obtained from SeV, overexpression of nsP1a/3 significantly and dose-dependently decreased the mRNA expression levels of IFN-β (Fig 8H), ISG15 (Fig 8I), and ISG56 (Fig 8J) induced by PAstV1-GX1, in comparison to the vector control (p < 0.05). Notably, the viral copy numbers of PAstV1-GX1 were significantly increased in groups overexpressing nsP1a/3 (p < 0.05; Fig 8K). To assess whether the IFN-antagonistic activity of nsP1a/3 is specific to certain cell types, HEK293T cells were transfected with the nsP1a/3 plasmid and subsequently infected with SeV. RT-qPCR analysis demonstrated that, contrary to the observations in PK-15 cells, nsP1a/3 overexpression in HEK293T cells did not significantly affect the mRNA expression levels of IFN-β (Fig 8L), ISG15 (Fig 8M), or ISG56 (Fig 8N) induced by SeV (p > 0.05), nor did it significantly influence SeV replication (p > 0.05) (Fig 8O).

To explore the potential inhibitory effects of nsP1a/3 on the production of biologically active type I IFN. PK-15 cells were transfected with varying concentrations of the nsP1a/3 plasmid or the pCAGGS vector (0.5, 1.5, or 2.5 µg) and subsequently infected with SeV. Ultraviolet (UV)-inactivated supernatants were then utilized to treat fresh PK-15 cells prior to infection with vesicular stomatitis virus expressing green fluorescent protein (VSV-GFP). Supernatants derived from SeV-infected control cells, which were demonstrated to contain elevated levels of IFN-β (S8 Fig), nearly completely inhibited VSV-GFP replication. In contrast, supernatants from cells overexpressing 1.5 µg of the nsP1a/3 plasmid significantly facilitated the restoration of VSV-GFP replication, as evidenced by a marked increase in the number of GFP-positive cells compared to the vector control. Notably, supernatants from cells expressing 2.5 µg of nsP1a/3 allowed VSV-GFP replication to reach levels comparable to those observed in the group infected with VSV-GFP alone (without SeV stimulation) (Fig 8P). Collectively, these findings demonstrate that the PAstV nsP1a/3 protein cleaves MAVS and effectively antagonizes type I IFN production, thereby suppressing both its transcription and the generation of biologically active IFN.

### The nsP1a/3-induced MAVS cleavage and type I IFN suppression occur independently of its apoptosis induction

**Fig 9:**
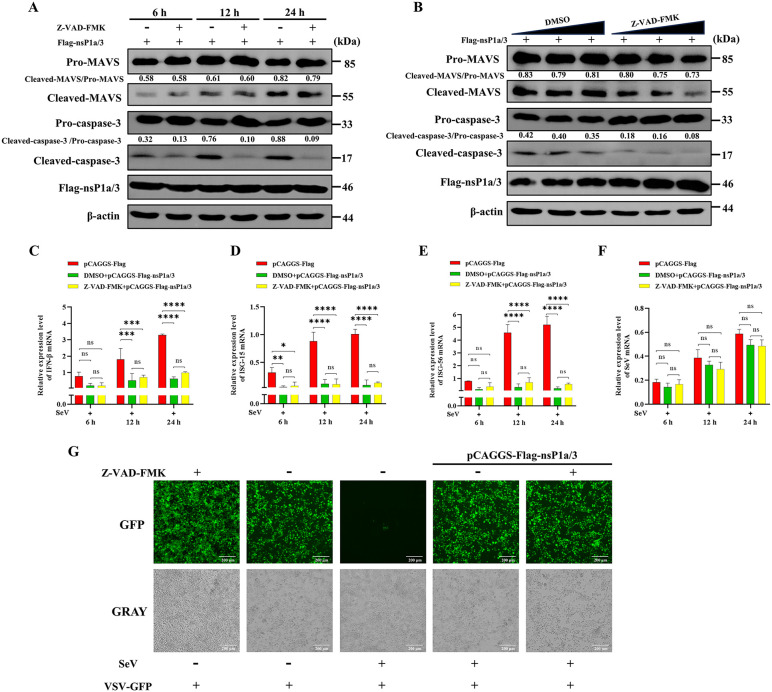
Inhibition of apoptosis does not affect nsP1a/3-induced MAVS cleavage and type I IFN suppression. **(A)** PK-15 cells were transfected with pCAGGS-Flag-nsP1a/3 and treated with or without 10 μM Z-VAD-FMK. Cell lysates were harvested at 24 hours post-transfection and subjected to Western blot analysis using antibodies against MAVS, caspase-3, Flag, and β-actin. The relative expression ratios of cleaved MAVS to pro-MAVS and cleaved caspase-3 to pro-caspase-3 were quantified by grayscale analysis using ImageJ software. **(B)** PK-15 cells transfected with pCAGGS-Flag-nsP1a/3 were treated with increasing concentrations of Z-VAD-FMK (5, 10, 15 µM) or DMSO as a control. Lysates were collected 24 hours post-transfection and analyzed by Western blot with antibodies specific for MAVS, caspase-3, Flag, and β-actin. The relative expression ratios of cleaved MAVS to pro-MAVS and cleaved caspase-3 to pro-caspase-3 were quantified based on grayscale values using ImageJ. (C, D, E, **F)** PK-15 cells transfected with either pCAGGS-Flag-nsP1a/3 or an empty vector were infected with SeV. The relative mRNA levels of porcine IFN-β **(C)**, ISG15 **(D)**, ISG56 **(E)**, and SeV (F) were measured by RT–qPCR at 6, 12, and 24 hpi, normalized to porcine β-actin mRNA. **(G)** PK-15 cells were mock-transfected or transfected with pCAGGS-Flag-nsP1a/3 and treated with or without Z-VAD-FMK. At 24 hours post-transfection, cells were infected with SeV for 12 hours. Supernatants were then collected, inactivated by UV irradiation, and applied to fresh PK-15 cells. After 24 hours of incubation, the recipient cells were infected with VSV-GFP, and viral replication was assessed by monitoring GFP fluorescence at 12 hpi. Data are presented as the mean ± SD from three independent experiments (n = 3). Statistical analysis was performed using two-way ANOVA, and significant differences compared to the empty vector control group are indicated as *p < 0.05, **p < 0.01, ***p < 0.001, and ****p < 0.0001.

To evaluate whether nsP1a/3 suppresses type I IFN signaling via the induction of apoptosis leading to the cleavage of MAVS, we transfected PK-15 cells with an nsP1a/3 expression plasmid and subsequently treated them with or without the pan-caspase inhibitor Z-VAD-FMK. Cell lysates were collected at 6, 12, and 24 hours post-transfection. Western blot analysis revealed that treatment with Z-VAD-FMK significantly reduced the levels of cleaved caspase-3 compared to the untreated control, thereby confirming the effective inhibition of nsP1a/3-induced apoptosis. However, this inhibition did not impact the cleavage of MAVS by nsP1a/3, as evidenced by the comparable levels of cleaved MAVS in cells treated with or without Z-VAD-FMK (Fig 9A). To further substantiate these findings, nsP1a/3-transfected PK-15 cells were treated with increasing concentrations of Z-VAD-FMK (5, 10, and 15 µM). Consistent with the initial observations, the levels of cleaved caspase-3 decreased in a dose-dependent manner. Importantly, the levels of cleaved MAVS remained unchanged and were similar to those observed in the nsP1a/3-transfected, DMSO-treated control group (Fig 9B).

To evaluate the influence of apoptosis on the suppression of type I IFN mediated by nsP1a/3, PK-15 cells transfected with either the nsP1a/3 plasmid or an empty vector were infected with SeV and treated with or without Z-VAD-FMK. RT-qPCR analysis revealed that overexpression of nsP1a/3 significantly reduced the SeV-induced expression of IFN-β (Fig 9C), ISG15 (Fig 9D), and ISG56 (Fig 9E) mRNA at 12 and 24 hpi compared to cells transfected with the empty vector (p < 0.001). Notably, treatment with Z-VAD-FMK did not significantly influence the expression of these genes in cells expressing nsP1a/3 (p > 0.05). Additionally, neither the overexpression of nsP1a/3 nor the treatment with Z-VAD-FMK significantly affected SeV mRNA levels (p > 0.05) (Fig 9F), suggesting that the observed suppression of IFN was not a consequence of altered viral replication.

To further substantiate the independence of IFN suppression from apoptosis, we conducted IFN bioassays utilizing VSV-GFP. The replication of VSV-GFP, quantified by GFP fluorescence, is inversely correlated with the levels of secreted type I IFN. Our findings revealed that overexpression of nsP1a/3 markedly inhibited type I IFN production, as evidenced by an increase in GFP fluorescence, indicative of enhanced VSV-GFP replication. Notably, treatment with Z-VAD-FMK did not significantly alter this inhibitory effect, as demonstrated by similar GFP fluorescence intensities in nsP1a/3-overexpressing cells, irrespective of the presence of the inhibitor (Fig 9G). Collectively, these results indicate that the apoptosis induced by nsP1a/3 does not influence its capacity to cleave MAVS or suppress type I IFN responses, thereby suggesting that these processes are distinct and independent.

### The catalytic triad of the 3C-like serine protease in PAstV nsP1a/3 is essential for MAVS cleavage, apoptosis induction, and type I IFN suppression

**Fig 10:**
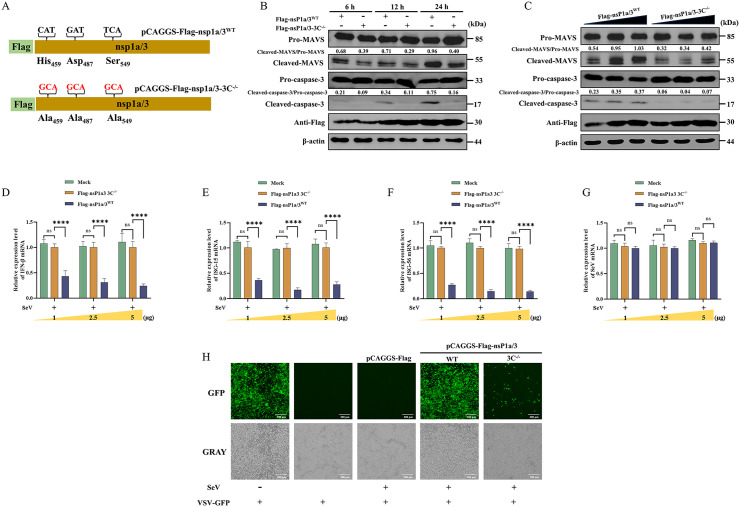
Effect of catalytic triad mutation in nsP1a/3 on MAVS cleavage, apoptosis and IFN-β induction. **(A)** Schematic representation of the mutagenesis of the serine protease catalytic triad (His_459_, Asp_487_, Ser_549_) in nsP1a/3 to alanine. The resulting mutant and wild-type plasmids were designated as pCAGGS-Flag-nsP1a/3-3C^-/-^ and pCAGGS-Flag-nsP1a/3^WT^, respectively. **(B)** PK-15 cells were transfected with either pCAGGS-Flag-nsP1a/3^WT^ or pCAGGS-Flag-nsP1a/3-3C^-/-^. Cell lysates were collected at 6, 12, and 24 hours post-transfection and subjected to Western blot analysis using antibodies against MAVS, caspase-3, Flag, and β-actin. The relative expression levels of cleaved MAVS to pro-MAVS and cleaved caspase-3 to pro-caspase-3 were quantified by grayscale analysis using ImageJ. **(C)** PK-15 cells were transfected with increasing doses (1, 2.5, and 5 μg) of pCAGGS-Flag-nsP1a/3^WT^ or pCAGGS-Flag-nsP1a/3-3C^-/-^. Lysates were harvested at 24 hours post-transfection and analyzed by Western blot with antibodies specific for MAVS, caspase-3, Flag, and β-actin. The ratios of cleaved MAVS to pro-MAVS and cleaved caspase-3 to pro-caspase-3 were quantified based on grayscale values using ImageJ. (D, E, F, **G)** PK-15 cells transfected with pCAGGS-Flag-nsP1a/3^WT^ or pCAGGS-Flag-nsP1a/3-3C^-/-^ were infected with SeV. The relative mRNA levels of porcine IFN-β **(D)**, ISG15 **(E)**, ISG56 **(F)**, and SeV (G) were measured by RT–qPCR at 6, 12, and 24 hpi and normalized to porcine β-actin mRNA. **(H)** PK-15 cells were either mock-transfected or transfected with pCAGGS-Flag-nsP1a/3^WT^, pCAGGS-Flag-nsP1a/3-3C^-/-^, or empty vector plasmid. At 24 hours post-transfection, cells were infected with SeV for 12 hours. Culture supernatants were collected, inactivated by UV irradiation, and applied to fresh PK-15 cells. After 24 hours, recipient cells were infected with VSV-GFP. Viral replication was assessed by monitoring GFP fluorescence at 12 hpi. Data are presented as mean ± SD from three independent experiments (n = 3). Statistical analysis was performed using two-way ANOVA. Statistical significance compared to the empty vector control group is indicated as ****p < 0.0001.

The nsP1a/3 protein of PAstV encompasses a 3C-like serine protease domain. To elucidate whether the 3C-like serine protease activity contributes to nsP1a/3-mediated apoptosis and MAVS cleavage, we employed site-directed mutagenesis to substitute the entire catalytic triad (His_459_, Asp_487_, and Ser_549_) within this domain with alanine (Ala) residues. The resultant mutant plasmid was designated as pCAGGS-Flag-nsP1a/3-3C^-/-^ (Fig 10A). Subsequently, PK-15 cells were separately transfected with either the wild-type plasmid pCAGGS-Flag-nsP1a/3^WT^ or the mutant plasmid pCAGGS-Flag-nsP1a/3-3C^-/-^. Western blot analysis indicated that, relative to the wild-type transfected group, cells transfected with the mutant plasmid exhibited significantly diminished levels of cleaved MAVS and cleaved caspase-3 at 6, 12, and 24 hours post-transfection (Fig 10B). To further validate these findings, PK-15 cells were transfected with increasing concentrations (1, 2.5, and 5 μg) of either pCAGGS-Flag-nsP1a/3^WT^ or pCAGGS-Flag-nsP1a/3-3C^-/-^. Western blot analysis revealed a dose-dependent increase in the levels of cleaved MAVS and cleaved caspase-3 in the wild-type group. Conversely, the mutant plasmid group exhibited no significant alterations in cleaved MAVS or cleaved caspase-3 levels across the tested doses, with these levels remaining significantly lower than those observed in the wild-type group (Fig 10C). To further explore the impact of these key residue mutations on the ability of nsP1a/3 to inhibit type I IFN, PK-15 cells transfected with either pCAGGS-Flag-nsP1a/3^WT^ or pCAGGS-Flag-nsP1a/3-3C^-/-^ were subsequently infected with SeV. RT-qPCR analysis indicated that, compared to the empty vector control, the expression of wild-type nsP1a/3 significantly suppressed SeV-induced mRNA expression of IFN-β (Fig 10D), ISG15 (Fig 10E), and ISG56 (Fig 10F) at 6, 12, and 24 hpi. Conversely, the catalytic triad mutant nsP1a/3 protein did not exhibit this suppressive effect, as the mRNA levels of IFN-β, ISG15, and ISG56 were comparable to those observed in the empty vector control group. Furthermore, transfection with either the pCAGGS-Flag-nsP1a/3^WT^ or pCAGGS-Flag-nsP1a/3-3C^-/-^ plasmid was not found to influence SeV replication (Fig 10G). An IFN bioassay provided further validation of these results, demonstrating that supernatants obtained from cells transfected with pCAGGS-Flag-nsP1a/3-3C^-/-^ following SeV infection significantly inhibited VSV-GFP replication. This was evidenced by GFP fluorescence signals that were markedly reduced compared to those observed in cells transfected with pCAGGS-Flag-nsP1a/3. These observations indicate that the catalytic triad mutant nsP1a/3 protein has largely lost its ability to suppress type I IFN. Collectively, these findings highlight the essential role of the catalytic triad residues (His_459_, Asp_487_, and Ser_549_) in the 3C-like serine protease of PAstV nsP1a/3 in mediating MAVS cleavage, apoptosis induction, and type I IFN suppression. Furthermore, amino acid sequence alignment revealed that these catalytic triad residues are highly conserved among astroviruses from various species (S9 Fig), implying that the immune- and apoptosis-modulating functions associated with this protease are likely conserved across diverse astroviral species.

### Inhibition of 3C-like serine protease activity suppresses PAstV replication

**Fig 11:**
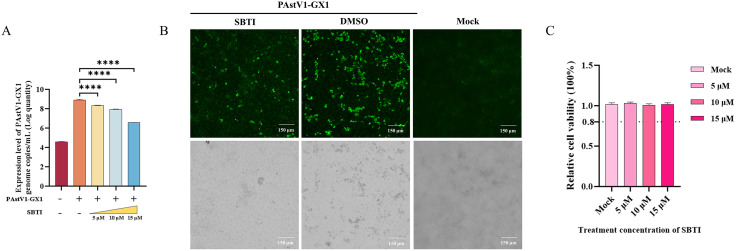
Serine protease inhibitor treatment suppresses PAstV replication. **(A)** PK-15 cells infected with PAstV1-GX1 (MOI = 0.1) were treated with increasing concentrations (5, 10, and 15 μM) of SBTI. Viral RNA copy numbers in cell lysates were quantified by RT-qPCR at 24 hpi. **(B)** PK-15 cells were either mock-infected or infected with PAstV1-GX1 (MOI = 0.1), followed by treatment with 15 μM SBTI or DMSO (control). Viral replication was assessed via IFA using a mouse anti-nsP1a/4 polyclonal antibody and an FITC-conjugated goat anti-mouse IgG secondary antibody. **(C)** Cell viability of PK-15 cells treated with SBTI (5, 10, and 15 μM) was evaluated using a CCK-8 assay. The data are presented as mean ± SD from three independent experiments (n = 3). Statistical analysis was performed using one-way ANOVA. ****p < 0.0001 indicates a statistically significant difference compared to the control group.

To examine the impact of inhibiting 3C-like serine protease activity on the replication of PAstV, PK-15 cells were infected with the PAstV1-GX1 strain and subsequently treated with escalating concentrations (5, 10, and 15 μM) of Soybean Protease Inhibitor (SBTI). At 24hpi, the cells were collected, and viral copy numbers were quantified using RT-qPCR. The findings indicated that SBTI treatment significantly inhibited PAstV replication in a dose-dependent manner (Fig 11A). To further substantiate the inhibitory effect, PK-15 cells infected with PAstV1-GX1 were exposed to 15 μM SBTI and analyzed by IFA at 24 hpi. A notable reduction in green fluorescence signal intensity was observed in the SBTI-treated group compared to the untreated control (Fig 11B), corroborating the potent antiviral efficacy of SBTI against PAstV. Additionally, cell viability assays revealed that treatment with 5, 10, and 15 μM SBTI did not result in significant cytotoxicity (Fig 11C), suggesting that the observed suppression of viral replication was not attributable to diminished cell viability but was likely due to specific inhibition of the 3C-like serine protease activity of PAstV.

## Discussion

Astroviruses are characterized by their extensive host range. Since their initial discovery in infant diarrheal stool samples in 1975 [18], these viruses have been identified in a wide array of mammalian and avian species, leading to a variety of clinical manifestations. HAstV infections, for instance, predominantly result in diarrhea and encephalitis among children and the elderly and are currently recognized as one of the top three causative agents of pediatric diarrhea [19]. Goose astrovirus, an emerging pathogen in recent years, poses a significant threat to the goose industry by primarily inducing gout in goslings, thereby causing substantial economic losses [20]. PAstV is highly prevalent in swine populations globally and is capable of causing diarrhea and encephalitis in suckling piglets [7,21], thereby jeopardizing the healthy development of the pig industry. Despite the broad host range and significant impact of astroviruses, research into their pathogenic mechanisms, particularly regarding virus-host cell interactions, remains limited and insufficiently comprehensive. This research gap significantly impedes the development of effective antiviral therapeutics against astroviruses. Therefore, elucidating the molecular mechanisms by which astroviruses manipulate host cellular networks to facilitate their own replication is crucial for understanding viral pathogenesis and developing novel antiviral strategies.

Apoptosis serves as a crucial host defense mechanism; however, certain viruses have evolved to exploit this process to facilitate their replication. Initial advancements have been made in elucidating the induction of apoptosis by HAstV[11]and GAstV[12], with evidence indicating a strong association between apoptosis and astrovirus replication and pathogenicity. Nonetheless, the precise mechanisms by which astroviruses induce apoptosis remain unclear. Importantly, there is a lack of studies examining the regulation of apoptosis by PAstV. In this study, we utilized the PAstV1-GX1 strain, previously isolated in our laboratory [21], as the research model. Our study shows that PAstV1-GX1 infection triggers significant apoptosis in PK-15 cells, with late apoptotic cells reaching 40% at 24 hpi. However, considering the viral replication kinetics, we hypothesize that the observed 40% apoptosis rate at 24 hpi with an initial MOI of 0.1 may result from a combination of cell death in directly infected cells and in adjacent, uninfected cells. This bystander effect, which has been documented in other viral systems [22,23], may be mediated by pro-apoptotic signals, such as cytokines and damage-associated molecular patterns (DAMPs), released from infected cells or through direct cell-to-cell contact. Nonetheless, the occurrence of bystander apoptosis during PAstV infection and the specific mechanisms involved require further investigation. TEM analysis revealed typical apoptotic changes, such as nuclear condensation and chromatin margination, and by 24 hpi, apoptotic bodies with many electron-dense viral particles had formed. Existing research suggests that the formation of apoptotic bodies is intricately linked to the dissemination of pathogens *in vivo*. For instance, cells infected with *Mycobacterium tuberculosis* produce apoptotic bodies that transport mycobacterial antigens to dendritic cells (DCs), thereby facilitating the intercellular spread of the bacterium [24]. Similarly, the African swine fever virus (ASFV) triggers apoptosis in porcine alveolar macrophages (PAMs) late in infection via the caspase-3 pathway, creating apoptotic bodies filled with viral particles. These bodies are then engulfed by nearby macrophages, leading to secondary infection [25]. Our findings indicate that PAstV infection of PK-15 cells also leads to the production of apoptotic bodies containing viral particles, strongly suggesting that PAstV may exploit this mechanism to enhance its replication. Nonetheless, the precise mechanisms underlying this process require further investigation.

Mitochondria are integral to the initiation of apoptosis, particularly in response to viral infections, which elicit intracellular stress signals that enhance mitochondrial membrane permeability and facilitate the release of mitochondrial constituents. These constituents subsequently activate caspase-9, ultimately leading to apoptosis [26]. Our research has verified that PAstV infection activates caspase-9 and caspase-3, while caspase-8 remains unaffected, suggesting that PAstV predominantly induces apoptosis via the intrinsic mitochondrial pathway. This finding contrasts with the results of Guix et al. [11], who reported that HAstV infection triggers apoptosis in CaCo-2 cells via caspase-8 activation. We propose that this discrepancy arises from the fact that HAstV and PAstV belong to distinct astrovirus species, characterized by low genetic homology and significant differences in biological properties. Furthermore, the two studies employed different culture systems. Our additional analyses have revealed that PAstV infection results in substantial mitochondrial damage, as evidenced by mitochondrial vacuolization, depolarization of the mitochondrial membrane potential, and the release of cytochrome c. These observations strongly imply that PAstV replication is closely associated with host cell mitochondria. Indeed, our most recent report has demonstrated that PAstV infection promotes its own replication by upregulating mitochondrial reactive oxygen species (ROS) levels [27].

Apoptosis is often intricately associated with the viral replication cycle, with certain viruses modulating apoptotic pathways to enhance their replication. For example, the gM protein of the pseudorabies virus facilitates viral replication by inducing apoptosis via the mitochondrial pathway [28], while the influenza virus promotes viral release by triggering apoptosis in the later stages of infection [29]. In our research, the pharmacological inhibition of apoptosis using Z-VAD-FMK markedly reduced PAstV replication at both transcriptional and translational levels. Conversely, the induction of apoptosis with ABT-263 during PAstV infection augmented viral replication at the translational level, although it did not significantly affect transcriptional activity. We propose that this discrepancy may be due to the nature of PAstV as a single-stranded positive-sense RNA virus, whose genome can directly serve as mRNA for viral protein synthesis. Once viral proteins are produced, apoptotic caspases may facilitate virion release—a mechanism well-documented in HAstV, where caspase-mediated cleavage of the capsid precursor protein VP90 to VP70 is essential for particle release [30]. Importantly, our team’s previous research demonstrated that PAstV-GX1 utilizes a distinct release mechanism. In contrast to HAstV, PAstV-GX1 is released from PK-15 cells without the prerequisite intracellular cleavage of VP90, thereby circumventing the conventional “VP90→VP70” processing pathway. Despite this, caspase activity still enhances viral egress, indicating a non-proteolytic, supportive role in particle release [31]. However, the induction of apoptosis could potentially impede the efficiency of viral genome transcription, as the cell progresses towards death or experiences a gradual degradation of the nuclear environment, such as chromatin condensation, thereby hindering a concurrent increase in transcriptional activity. To further elucidate this phenomenon, we administered ABT-263 at 6, 12, and 18 hpi. Our observations revealed that the addition of ABT-263 at 12 and 18 hpi led to a modest but statistically significant increase in viral replication, whereas administration at 6 hpi did not produce a notable effect. This suggests that caspase-mediated apoptosis during the late stages of PAstV infection may contribute to viral replication, potentially by facilitating viral release.

The nsP1a polyprotein consists of an N-terminal protein (nsP1a/1), a highly hydrophobic protein (nsP1a/2), a 3C-like serine protease (nsP1a/3), and nsP1a/4, which is hypothesized to be processed into a viral protein genome-linked (VPg) and a hypervariable region [32]. Through flow cytometry and Western blot analysis, nsP1a/3 has been identified as the principal viral protein responsible for PAstV-induced apoptosis. This protein interacts with MAVS and is localized to the mitochondria. Silencing MAVS expression suppressed PAstV-induced apoptosis and enhanced PAstV replication during the early stages of infection (6 and 12 hours post-infection), but inhibited replication at later stages (≥24 hours post-infection), as indicated by significantly reduced viral titers in the supernatant of MAVS-knockdown cells compared to wild-type cells (p < 0.01). This observation further implies that the suppression of apoptosis via MAVS knockdown hinders the release of PAstV. The observed increase in early replication in MAVS-knockdown cells is likely attributable to the reduced production of type I IFN, which fosters a more conducive environment for initial viral replication. In contrast, the overexpression of MAVS significantly augmented PAstV-induced apoptosis while substantially inhibiting PAstV replication. This apparent contradiction with our finding that induced apoptosis promotes PAstV replication, as observed with ABT-263, can be elucidated by two factors: (1) MAVS overexpression induces robust type I IFN production, which strongly inhibits viral replication; (2) as obligate intracellular parasites, viruses are heavily reliant on living host cells for resources and energy. The overexpression of MAVS triggers rapid apoptosis early in the infection process, leading to accelerated CPE and premature cell death, thereby depriving PAstV of adequate time to produce progeny virions. This is corroborated by IFA results, which confirmed that MAVS overexpression led to significant cell detachment.

It is well established that MAVS serves as a critical platform for type I IFN signaling, and numerous viruses target MAVS to circumvent this antiviral response. Unexpectedly, we discovered that the nsP1a/3 protein cleaves MAVS in a dose-dependent manner, thereby inhibiting type I IFN production. Notably, the inhibition of apoptosis using Z-VAD-FMK did not influence MAVS cleavage or the suppression of type I IFN, indicating that the pro-apoptotic and type I IFN antagonistic functions of nsP1a/3 are distinct processes. The nsP1a/3 protein exhibits 3C-like serine protease activity, which typically relies on a catalytic triad composed of serine (Ser), histidine (His), and aspartic acid (Asp) residues. Site-directed mutagenesis of these residues, which disrupts the protease activity, significantly impaired the ability of nsP1a/3 to activate caspase-3 and cleave MAVS. This finding establishes that the 3C-like serine protease activity of nsP1a/3 is essential for both inducing apoptosis and suppressing the type I IFN response. Nevertheless, the catalytic triad mutant (3C^−/−^) still produced a reduced yet detectable level of cleaved MAVS, indicating that either residual protease activity or alternative, 3C-independent mechanisms—such as the recruitment of other host factors—might contribute to MAVS processing. Previous research has documented the role of viral proteases in regulating apoptosis and antagonizing type I IFN. For example, the 3CL^pro^ of the porcine epidemic diarrhea virus (PEDV) induces mitochondrial damage and MAVS-mediated apoptosis [33], while the 3C protease of the porcine reproductive and respiratory syndrome virus (PRRSV) cleaves MAVS to inhibit IFN-β expression [34]. To our knowledge, this study is the first to demonstrate that the PAstV nsP1a/3 protein exhibits a functional dichotomy—inducing apoptosis and antagonizing type I IFN—both of which are critically dependent on its 3C-like serine protease activity. Notably, our previous study demonstrated that the PAstV nsP1a/4 protein antagonizes the type I IFN response, whereas the nsP1a/3 protein does not [16]. This discrepancy can be attributed to the use of different cell lines: the earlier experiments were conducted in human HEK293T cells, while the present study utilized porcine PK-15 cells. Notably, in the current work, we also observed that overexpression of nsP1a/3 in HEK293T cells failed to suppress SeV-induced type I IFN production, confirming that the inhibitory effect of PAstV nsP1a/3 on type I IFN signaling is species-specific. This specificity is likely attributable to the relatively low amino acid identity (54.3%) between swine and human MAVS (S10 Fig), which may influence the recognition or cleavage efficiency of nsP1a/3.

**Fig 12:**
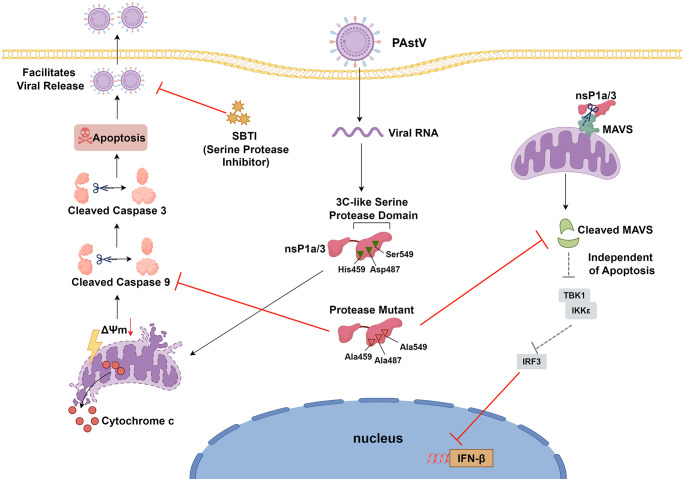
The model illustrates the dual function of PAstV nsP1a/3 in regulating apoptosis and innate immunity. (By Figdraw.com). Upon invasion of host cells by PAstV, the nsP1a/3 protein plays a dual role. Firstly, it activates caspase 9 and caspase 3, leading to apoptosis and facilitating viral replication through the mitochondrial pathway. Secondly, the nsP1a/3 protein cleaves MAVS, thereby inhibiting the production of type I IFN. Mutation of the serine protease catalytic triad in nsP1a/3 to alanine significantly reduces its ability to induce apoptosis and suppress type I IFN production. Additionally, treatment with soybean trypsin inhibitor (SBTI) has been shown to inhibit PAstV replication.

In conclusion, this study represents the first to elucidate that PAstV infection in PK-15 cells triggers apoptosis via the mitochondrial pathway, a process that facilitates viral replication. Further investigations have identified the nsP1a/3 protein as a crucial factor in PAstV-induced apoptosis, demonstrating its localization to mitochondria through interaction with the MAVS protein. Concurrently, nsP1a/3 expression results in MAVS cleavage and suppression of the type I IFN response. Mutagenesis disrupting the 3C-like serine protease activity within the nsP1a/3 protein significantly diminishes its capacity to induce apoptosis, cleave MAVS, and inhibit the type I IFN response. Moreover, the application of serine protease inhibitors markedly impedes PAstV replication (Fig 12) This study provides critical insights into the pathogenic mechanisms of PAstV and informs the development of antiviral therapeutics.

## Materials and methods

### Cell lines and viruses

PK-15, HEK-293T, and BHK-21 cell lines were maintained in our laboratory. Cells were cultured in Dulbecco’s Modified Eagle Medium (DMEM; Gibco, MD, USA) supplemented with 10% fetal bovine serum (FBS; Gibco), 100 U/mL penicillin, and 100 μg/mL streptomycin at 37°C under 5%CO₂. The PAstV1-GX1 strain (GenBank accession No. KF787112) was isolated and preserved in our laboratory. VSV-GFP and SeV were obtained and stored in our facility.

### Antibodies and chemical reagents

Anti-caspase-3 (19677–1-AP), anti-caspase-8 (13423–1-AP), anti-caspase-9 (10380–1-AP), anti-cytochrome c (10993–1-AP), anti-VDAC1 (55259–1-AP), anti-β-actin (66009–1-Ig), anti-MAVS (66911–1-Ig), Multi-rAb CoraLite Plus 594-conjugated goat anti-mouse IgG (H + L) (RGAM004), CoraLite Plus 488-conjugated goat anti-rabbit IgG (H + L) (RGAR002), and HRP-conjugated goat anti-rabbit IgG(H + L) (SA00001–2) were purchased from Proteintech (Wuhan, China). FITC-conjugated goat anti-mouse IgG (GB22301), Recombinant anti-Flag rabbit monoclonal antibody (mAb) (GB15939), Recombinant anti-HA mouse mAb (GB151252) were purchased from Servicebio (Wuhan, China). Anti-PAstV1 nsP1a/3 and nsP1a/4 mouse polyclonal antibodies were produced in our laboratory. Reagents such as DAPI (P0131), Z-VAD-FMK (C1202), ABT-263 (SC0011), Soybean Protease Inhibitor (SG2031), and CCK-8 (C0037) were obtained from Beyotime Biotechnology (Shanghai, China).

### Plasmid

To generate eukaryotic expression plasmids for the PAstV capsid protein and the cellular mitochondrial proteins VDAC1 and VDAC2, total RNA was isolated from PAstV1-GX1-infected PK-15 cells using a total RNA extraction kit (Tiangen Biotech, Beijing, China), following the manufacturer’s instructions. First-strand cDNA synthesis was performed using a one-step reverse transcription kit (Takara, Dalian, China). Subsequently, the capsid protein, VDAC1, and VDAC2 were amplified via PCR with specific primers detailed in Table 1. Flag tag sequences were fused to the C-terminus of the capsid protein, while HA tag sequences were fused to the C-terminus of VDAC1 and VDAC2. The amplified fragments were cloned into the pCAGGS vector with specific restriction sites, resulting in plasmids designated as pCAGGS-Flag-capsid, pCAGGS-HA-VDAC1, and pCAGGS-HA-VDAC2, respectively. All constructs were validated by DNA sequencing. Additional eukaryotic expression plasmids for PAstV nsP1a/1 (pCAGGS-Flag-nsP1a/1), nsP1a/3 (pCAGGS-Flag-nsP1a/3), nsP1a/4 (pCAGGS-Flag-nsP1a/4), and MAVS (pCAGGS-HA-MAVS), as well as the luciferase reporter plasmid carrying the porcine IFN-β promoter (pIFN-β-Luc), had been previously constructed in our laboratory [17]. The mitochondria marker plasmid pDsRed2-Mito was purchased from Wuhan Miaoling Biotechnology Co., Ltd. (Wuhan, China).

**Table 1:** Sequences of the PCR primers used in this study.

Primer	Sequence (5’ to 3’)
Capsid-F	CG*AAGCTT*_(HindIII)_ATGGCTAGCAAGTCTGGCA
Capsid-R	CC*GGATCC*_(BamHI)_TTACTTATCGTCGTCATCCTTGTAATCCTCGGCGTGGCCTCGGCTT
VDAC1-F	CCG*CTCGAG*_(XhoI)_CGGATGGCTGTGCCACCCACGTAT
VDAC1-R	CTA*GCTAGC*_(NheI)_TAGTTAAGCGTAGTCTGGGACGTCGTATGGGTATGCTTGAAACTCCAGTCCTAGAC
VDAC2-F	GAT*CTCGAG*_(XhoI)_ATGGCGACCCACGGACAGACTTGC
VDAC2-R	CTA*GCTAGC*_(NheI)_TATAGCGTAGTCTGGGACGTCGTATGGGTATTAAGCCTCCAACTCCAGGGCA

Restriction enzyme sequences are shown in italics, and Flag or HA tag sequences are underlined.

### Analysis of virus-induced apoptosis by annexin V-FITC/PI staining and flow cytometry

PK-15 cells were cultured in 12-well plates and subsequently infected with the PAstV1-GX1 strain at a multiplicity of infection (MOI) of 0.1 once the cell confluence reached approximately 80%. Uninfected cells were concurrently maintained as negative controls. At 6, 12, and 24 hpi, cells were detached with EDTA-free trypsin and incubated at 37°C for 10 minutes. The cells underwent three washes with ice-cold phosphate-buffered saline (PBS), with each wash followed by centrifugation at 1000 × g for 5 minutes. After the final wash, the supernatant was removed, and the cells were sequentially stained with Annexin V-FITC and propidium iodide (PI) using the Annexin V-FITC/PI Apoptosis Detection Kit (Vazyme Biotech, Nanjing, China) according to the manufacturer’s protocol. Subsequently, 20 μL of the cell suspension was placed onto a sterilized glass slide and examined using a fluorescence microscope to assess cell staining at various time points. Early apoptotic cells were identified by green fluorescence (Annexin V^+^/PI^-^), whereas late apoptotic cells exhibited both green and red fluorescence (Annexin V^+^/PI^+^), resulting in a yellow appearance due to fluorescence overlap. In contrast, normal cells demonstrated negligible fluorescence (Annexin V-/PI-). To further quantify the extent of apoptosis induced by PAstV infection, the Annexin V-FITC/PI-stained PK-15 cell suspension was filtered through a 40-μm nylon mesh to achieve a single-cell suspension. The proportions of early and late apoptotic cells at each time point were quantified via flow cytometry. Data analysis was performed using FlowJo software (BD Bioscience).

### Transmission electron microscopy (TEM) observation

PK-15 cells, cultured in 6-well plates, were infected with the PAstV1-GX1 strain at an MOI of 0.1, while uninfected cells served as negative controls. At 6, 12, and 24 hpi, the cells were detached with 0.25% trypsin, followed by harvesting and three washes with ice-cold PBS. The resulting cell pellets were fixed overnight at 4°C in 2.5% glutaraldehyde. After three additional washes with ice-cold PBS, the pellets were post-fixed in 1% osmium tetroxide at 4°C for 1 hour. The samples were then dehydrated using a graded ethanol series (50%, 70%, 80%, 95%, 100%) and infiltrated with epoxy resin overnight. Polymerization was conducted at 60°C for 24 hours, after which ultrathin sections (~100 nm) were prepared. These sections were double-stained with uranyl acetate and lead citrate, each for 10 minutes at room temperature, and subsequently examined using a Hitachi HT-7700 transmission electron microscopy (TEM) to assess virus-induced morphological changes in the cells and mitochondria.

### Western blotting analysis

PK-15 cells were cultured in 6-well plates and subjected to either infection with the PAstV1-GX1 strain at an MOI of 0.1 or transfection with the specified plasmid utilizing the Lipo6000 transfection reagent (Beyotime). At designated intervals post-infection or transfection, the cells were lysed using RIPA lysis buffer (Solarbio, Beijing, China) supplemented with 1% phenylmethylsulfonyl fluoride (PMSF) (Solarbio). Total cellular proteins were extracted by centrifuging the lysates to obtain the supernatant. The protein samples were separated using 10% SDS-PAGE, with electrophoresis performed initially at 80 V for 30 min followed by 120 V for 90 min. Subsequently, proteins were transferred onto a polyvinylidene fluoride (PVDF) membrane (Merck Millipore, Darmstadt, Germany) at a constant current of 300 mA for 30 min. The membrane was then blocked with 5% (w/v) skim milk for 2 h at room temperature. Following incubation with specific primary and secondary antibodies, the target protein bands were visualized using the BeyoECL Plus kit (Beyotime) and imaged with a Bio-Rad ChemiDoc system. The intensity of the bands (gray-scale values) was quantified using ImageJ software (version 1.48; NIH).

### Mitochondrial/Cytosolic Fractionation and Cytochrome c Localization Analysis

PK-15 cells were infected with the PAstV1-GX1 strain at an MOI of 0.1. At 6, 12, and 24 hpi, the cells were detached using 0.25% trypsin and collected by centrifugation. Mitochondrial and cytosolic fractions were subsequently isolated using a Cell Mitochondria Isolation Kit (Beyotime), in accordance with the manufacturer’s protocol. Briefly, the harvested cells were gently resuspended in the mitochondria isolation reagent and incubated on ice for 15 minutes. The cell suspension was then transferred to an appropriately sized glass homogenizer and homogenized with 20 strokes. The homogenate was centrifuged at 600 × g for 10 minutes at 4°C. The supernatant was carefully transferred to a new microcentrifuge tube and subjected to further centrifugation at 11,000 × g for 10 minutes at 4°C. This procedure resulted in the separation of the supernatant (cytosolic fraction, devoid of mitochondria) and the pellet (mitochondria-enriched fraction). The mitochondrial pellet was lysed in mitochondrial lysis buffer supplemented with 1% PMSF for 10 minutes on ice. This was followed by centrifugation at 13,000 × g for 10 minutes at 4°C, after which the supernatant containing the mitochondrial protein extract was collected. Both cytosolic and mitochondrial protein extracts were subjected to SDS-PAGE and subsequently transferred onto PVDF membranes. The membranes were probed with a rabbit polyclonal anti-cytochrome c antibody as the primary antibody, followed by an HRP-conjugated goat anti-rabbit IgG antibody as the secondary antibody, to evaluate the expression dynamics of cytochrome c in each compartment. VDAC1 and β-actin served as loading controls for the mitochondrial and cytosolic fractions, respectively. Fraction purity was confirmed by absence of VDAC1 in cytosolic fractions and β-actin in mitochondrial fractions.

### Assessment of mitochondrial membrane potential

PK-15 cells were cultured in 96-well plates and subsequently infected with the PAstV1-GX1 strain at an MOI of 5. At specified time intervals (6, 12, 18, and 24 hpi), the mitochondrial membrane potential (ΔΨm) was evaluated utilizing a mitochondrial membrane potential assay kit provided by Beyotime Biotechnology, following the manufacturer’s protocol. The cells were stained with the JC-1 probe at a dilution of 1:200 and incubated at 37°C for 30 minutes. Post-incubation, the supernatant was aspirated, and the cells were washed twice with JC-1 staining buffer. Subsequently, 100 µL of DMEM was added to each well, and fluorescence analysis was conducted using a fluorescence microscope. A high ΔΨm facilitates the formation of J-aggregates within the mitochondrial matrix, which emit red fluorescence. In contrast, a low ΔΨm inhibits JC-1 aggregation, resulting in the presence of JC-1 monomers (J-monomers) that emit green fluorescence. ImageJ software was utilized to quantify the intensities of red and green fluorescence at each time point, and alterations in ΔΨm were determined by calculating the relative fluorescence density ratio (J-aggregates/J-monomer).

### Apoptosis modulation and PAstV replication

To assess the impact of apoptosis on PAstV replication, PK-15 cells cultured in 24-well plates were pretreated for 2 hours at 37°C with either the pan-caspase inhibitor Z-VAD-FMK (10 μM) or the apoptosis inducer ABT-263 (5 μM) prior to infection with PAstV1-GX1 at an MOI of 0.1. The concentrations of the inhibitor and inducer were maintained throughout the experimental period. At 6, 12, and 24 hpi, both cells and supernatants were collected. Total viral RNA was extracted from these samples and quantified using reverse transcription quantitative PCR (RT-qPCR) to determine viral RNA copy numbers, as previously described [17]. Concurrently, total cellular protein was extracted for Western blot analysis to detect the activation of caspase-3 (using an anti-caspase-3 antibody) and the expression of the PAstV nsP1a/4 protein (using an anti-nsP1a/4 antibody). To further explore the effect of apoptosis induction timing on viral replication, PK-15 cells were infected with PAstV1-GX1 (MOI = 0.1), and ABT-263 (5 μM) was subsequently added at 6, 12, or 18 hpi. Cells and supernatants were collected at 24 hpi, and viral RNA copy numbers in both fractions were quantified by RT-qPCR.

### Co-IP

For the co-immunoprecipitation (Co-IP) assay, HEK-293T cells at approximately 60% confluence were either co-transfected with the specified plasmid or infected with the PAstV1-GX1 strain. At 24 hours post-transfection, the cells were lysed using IP lysis buffer (Solarbio). BeyoMag Protein A + G magnetic beads (Beyotime) were separately conjugated with anti-Flag, anti-HA, or anti-MAVS antibodies. Subsequently, 500 μL of each cell lysate was incubated with 20 μL of the antibody-conjugated beads at 4°C for 12 hours with continuous rotation. The bead complexes were isolated magnetically and subjected to five washes with IP lysis buffer. Proteins bound to the beads were eluted by boiling in 1 × SDS-PAGE loading buffer for 10 minutes. The eluted proteins, along with the corresponding whole cell lysate (WCL) samples, were resolved by SDS-PAGE and analyzed via Western blotting. Immunoblotting was conducted using anti-Flag, anti-HA, anti-nsP1a/3, or anti-MAVS antibodies to detect the respective proteins.

### Confocal microscopy analysis

BHK-21 cells were cultured in 35 mm confocal dishes. Once the cells reached approximately 60% confluence, the specified eukaryotic expression plasmids were co-transfected into the cells. At 24 hours post-transfection, the cells were fixed with 4% paraformaldehyde and incubated at 4°C for 30 minutes. Following fixation, the cells were blocked with 1% BSA at 37°C for 2 hours. The cells were then incubated with either the anti-Flag rabbit mAb or anti-HA mouse mAb at 37°C for 3 hours. After five washes with PBS, the cells were incubated with the CoraLite Plus 488-conjugated goat anti-rabbit IgG or Multi-rAb CoraLite Plus 594-conjugated goat anti-mouse IgG secondary antibody for 1 hour at 37°C. Subsequently, following an additional five PBS washes, the cells were stained with 4’,6-diamidino-2-phenylindole (DAPI; Solarbio). The expression and subcellular localization of the target proteins were visualized using a Zeiss LSM 310 confocal laser scanning microscope (Zeiss, Oberkochen, Germany). Co-localization analysis was conducted using ImageJ software.

### Structural Prediction, Molecular Docking, and Interaction Analysis of nsP1a/3 and MAVS

The tertiary structures of the nsP1a/3 protein from the PAstV1-GX1 strain (GenBank accession no. NC025379) and porcine MAVS (GenBank accession no. MK302496) were predicted utilizing the AlphaFold 3 online platform (https://golgi.sandbox.google.com). Visualization and editing of the predicted structures were performed using PyMOL software. To explore potential interaction regions between nsP1a/3 and MAVS, a molecular docking approach was employed, adhering to established methodologies. Initially, the HDOCK server facilitated the generation of preliminary conformational models of the protein complex. These models underwent refinement via the Rosetta docking protocol, resulting in an ensemble of complex structures. The models were subsequently screened and clustered based on Rosetta energy scoring functions, with the model exhibiting the lowest energy score being selected as the optimal complex structure, indicative of the native binding mode. Finally, protein–protein interactions between nsP1a/3 and MAVS were analyzed at the atomic level using the Protein–Ligand Interaction Profiler (PLIP) algorithm.

### Indirect Immunofluorescence Assay (IFA)

For the immunofluorescence assay (IFA), PK-15 cells were infected with the PAstV1-GX1 strain at an MOI of 1. At 6, 12, and 24 hpi, the cells were fixed using 4% paraformaldehyde. Subsequently, they were incubated with anti-PAstV1 nsP1a/4 mouse polyclonal antibodies at 37°C for 1 hour. Following five washes with PBS, the cells were incubated with a FITC-conjugated goat anti-mouse IgG secondary antibody for 1 hour at 37°C. After an additional five washes with PBS, the cells were examined using a fluorescence microscope (Nikon, Tokyo, Japan).

### siRNA knockdowns and plasmid overexpression

The siRNA specific to Sus scrofa MAVS (siMAVS; sense strand: 5′-GACAAGACUUAUCAGUAUA-dTdT-3′; antisense strand: 5′-UAUACUGAUAAGUCUUGUC-dTdT-3′) was designed and synthesized by Sangon Biotech (Shanghai, China). For experiments involving siRNA-mediated knockdown or plasmid-mediated overexpression, PK-15 cells were cultured in 6-well plates. Upon achieving approximately 70% confluency, the cells were transfected with either siMAVS or the pCAGGS-HA-MAVS plasmid utilizing the Lipo6000 transfection reagent (Beyotime), following the manufacturer’s protocol. Transfection with a non-targeting siRNA control (siNC) or the empty pCAGGS-HA vector was employed as the negative control. The efficiency of MAVS knockdown or overexpression was evaluated through Western blot analysis at various time points post-transfection.

### CCK8 assay

Cell viability was assessed using a CCK8 assay kit (Beyotime). PK-15 cells were seeded at a density of 10,000 cells per well into 96-well plates and incubated overnight. Subsequently, the cells were treated with the respective compounds or transfected with siRNA for 24 hours. The CCK8 reagent was then applied according to the manufacturer’s instructions, and absorbance was measured at 490 nm (OD 490) using a plate reader. All experiments were performed in triplicate (n = 3 per experiment) and independently replicated twice.

### Multistep growth curves

To evaluate the in vitro replication kinetics of the PAstV1-GX1 strain in MAVS-knockdown PK-15 cells compared to wild-type PK-15 cells, both cell types were inoculated with the PAstV1-GX1 strain at an MOI of 0.01. Samples of cells and supernatants were collected at 6, 12, 18, 24, 36, 48, 60, and 72 hpi. The viral titers in these samples were quantified using a 50% tissue culture infectious dose (TCID₅₀) assay conducted on PK-15 cells, with calculations performed according to the Reed and Muench method [35]. Subsequently, viral growth curves were generated, plotting infection time on the x-axis and virus titer on the y-axis.

### Relative quantitative Real-time PCR

Total RNA was extracted from PK-15 cells using the Total RNA Extraction Kit (Tiangen), following the manufacturer’s protocol meticulously. For RT-qPCR, cDNA was synthesized using the One-Step Reverse Transcription Kit (Takara). Quantitative PCR was performed with the ChamQ Universal SYBR qPCR Master Mix (Vazyme Biotech). Gene expression levels were normalized to the expression of the housekeeping gene β-actin. Each experiment was replicated at least three times, and relative mRNA expression levels were determined using the 2^(−ΔΔCt) method. The relative quantitative PCR primer sequences employed in this study were previously designed in our earlier research [17] and are detailed in S1 Table.

### Site-directed mutagenesis

Site-directed mutagenesis was conducted utilizing the QuickMutation Kit (Beyotime) in accordance with the manufacturer’s protocol, employing primers detailed in S2 Table. The PCR assays, with a total volume of 50 μL, incorporated the kit components and 200 ng of the template plasmid (pCAGGS-Flag-nsP1a/3). The thermal cycling parameters were as follows: initial denaturation at 95°C for 3 minutes; 20 cycles of denaturation at 95°C for 30 seconds, annealing at 55°C for 30 seconds, and extension at 68°C for 30 seconds; followed by a final extension at 68°C for 10 minutes. The PCR products were subjected to DpnI digestion at 37°C for 5 minutes, and subsequently transformed into DH5α cells. Screening of individual colonies was performed via PCR, and the presence of desired mutations was confirmed through sequencing. Successive rounds of mutagenesis, using the resultant mutant plasmids as templates, facilitated the substitution of all three residues (His_459_, Asp_487_, Ser_549_) of the nsP1a/3 3C-like serine protease catalytic triad with alanine. The final construct was designated as pCAGGS-Flag-nsP1a/3-3C^-/-^.

### Dual luciferase reporter assay

The dual-luciferase reporter assay was performed as previously described [17]. Briefly, PK-15 cells were cultured in 24-well plates and allowed to reach approximately 70% confluence. The cells were then co-transfected with 1 μg of pCAGGS-Flag-nsP1a/3, 350 ng of pIFN-β-Luc, and 50 ng of pRL-TK (Promega, USA). After 24 hours post-transfection, the cells were infected with SeV at a dose of 10 hemagglutinating activity units (HAU) per well for a duration of 12 hours. Following this incubation period, the activities of firefly luciferase and Renilla luciferase were quantified in the cell lysates using a dual-luciferase reporter assay kit (Servicebio). Data from three independent experiments are presented as the ratio of firefly luciferase activity to Renilla luciferase activity.

### ELISA for porcine IFN-β detection

PK-15 cells cultured in 24-well plates were infected with SeV (10 HAU/well) at ~90% confluence. Cell culture supernatants were harvested at 18 hpi. The concentration of porcine IFN-β in the supernatants was quantified using a commercially available porcine IFN-β-specific enzyme-linked immunosorbent assay (ELISA) kit (Solarbio), following the manufacturer’s protocol. In brief, standards and samples were introduced into a 96-well plate pre-coated with the specific antibody and incubated at 37°C for 30 minutes. Post-incubation, the wells were aspirated and washed with PBS, after which a HRP-conjugated detection antibody was added, and the plate was incubated at 37°C for an additional 30 minutes. Subsequently, the substrate TMB was added to each well to facilitate color development. The optical density (OD) at 450 nm was determined using a microplate reader. A standard curve was constructed from the OD values of the standards, and the concentration of each sample was calculated using the curve equation.

### IFN bioassay

PK-15 cells were cultured in 6-well plates and transfected with increasing concentrations (0.5, 1.5, and 2.5 μg) of either the pCAGGS-Flag-nsP1a/3 plasmid or the control empty vector pCAGGS. Twenty-four hours post-transfection, the cells were inoculated with SeV at a dose of 10 HAU per well. Cell supernatants were collected at 18 hpi and subjected to ultraviolet (UV) irradiation for 3 hours to inactivate any remaining infectious SeV. The UV-irradiated supernatants were then applied at a volume of 1 mL per well to fresh monolayers of PK-15 cells. After a 24-hour incubation period, the supernatant was removed, and the cells were infected with the interferon-sensitive reporter virus VSV-GFP at an MOI of 1. The replication of VSV-GFP was evaluated using fluorescence microscopy at 18 hours post-VSV infection.

### Statistical analysis

All experiments were conducted with a minimum of three independent replicates (n ≥ 3). Statistical analyses were executed utilizing GraphPad Prism version 6.0. For intergroup comparisons, appropriate statistical tests, including the one-way analysis of variance (ANOVA) or two-way ANOVA, were employed. Data derived from at least three independent experiments are presented as the mean ± standard deviation. Statistical significance is denoted in the figures as follows: *, p < 0.05; **, p < 0.01; ***, p < 0.001and ****, p < 0.0001.

## Supporting information

S1 Fig PK-15 cells were infected with PAstV1-GX1 (MOI = 0.1) and simultaneously treated with or without 5 μM ABT-263. Intracellular (A) and extracellular (B) viral RNA levels at the indicated time points post-infection were quantified by RT-qPCR. Data are presented as mean ± SD from three independent experiments (n = 3). Statistical analysis was performed using two-way ANOVA; no significant differences were found between the ABT-263-treated and control groups at any time point.
(DOCX)

S2 Fig HEK-293T cells were co-transfected with plasmids for pCAGGS-Flag-nsP1a/3 and either pCAGGS-HA-VDAC1 (A, B) or pCAGGS-HA-VDAC2 (C, D) for 24 hours. Cell lysates were subjected to co-immunoprecipitation (co-IP) with anti-Flag or anti-HA beads. The precipitated proteins, along with whole-cell lysates (WCL), were analyzed by Western blot using anti-HA and anti-Flag antibodies. β-Actin was used as a loading control.
(DOCX)

S3 Fig (A) Schematic representation of the locations of relevant structural regions within the PAstV nsP1a polyprotein. The 3C-like serine protease region located in the nsP1a/3 is highlighted. (B) Docking complex model of nsP1a/3-MAVS constructed using the AlphaFold 3 engine and PLIP docking algorithm.
(DOCX)

S4 Fig HEK-293T cells were co-transfected with plasmids expressing Flag-tagged nsP1a/4 and HA-tagged MAVS for 24 h. Cell lysates were subjected to co-immunoprecipitation (co-IP) using anti-Flag or anti-HA magnetic beads to assess the interaction. Immunoprecipitates and whole-cell lysates (WCL, input) were then analyzed by western blotting with anti-HA and anti-Flag antibodies, respectively. β-actin served as a loading control.
(DOCX)

S5 Fig Cell v iability assessed by CCK-8 assay in PK-15 cells transfected with graded doses (0.5, 1.0, 2.5, and 5 μg) of siMAVS at 24 hours post-transfection. (DOCX)

S6 Fig Quantification of fluorescence intensity across different treatment groups was performed using ImageJ software. Data are expressed as mean ± SD from three independent experiments (n = 3). Statistical significance was assessed by two-way ANOVA. Significant differences compared to the siNC group are denoted as *p < 0.05 and **p < 0.01.
(DOCX)

S7 Fig PK-15 cells were transfected with increasing amounts of the pCAGGS-Flag-nsP1a/4 plasmid, using untransfected cells as negative controls (Mock). Cell lysates were collected at 24 h post-transfection and analyzed by Western blot using antibodies against MAVS, Flag, and β-actin.
(DOCX)

S8 Fig The concentration of porcine IFN‑β in supernatants from SeV‑infected PK‑15 cells was determined using a porcine IFN‑β‑specific ELISA. Mock represents the uninfected control. Data are expressed as mean ± SD from three independent experiments (n = 3). Statistical significance was assessed by unpaired t‑test. Significant differences compared to the mock group are denoted as ****p < 0.0001.
(DOCX)

S9 Fig Alignment of the catalytic triad residues in the 3C-like serine protease across different astrovirus species. The catalytic triad residues are highlighted in dark color.
(DOCX)

S10 Fig Full-length amino acid sequence alignment of porcine and human MAVS proteins. The full-length mRNA sequences of porcine MAVS (GenBank accession no. MK302496.1) and human MAVS (GenBank accession no. KC415005.1) were downloaded from the NCBI database and aligned using the MegAlign software.
(DOCX)

S1 Table Analysis of docking interactions between nsP1a/3 and MAVS. (DOCX)

S2 Table The relative quantitative PCR primers used in this study. (DOCX)
