# Supplementary material for: The 3C-like serine protease activity of porcine astrovirus nsP1a/3 mediates mitochondrial apoptosis and MAVS cleavage to facilitate viral replication and antagonize type I interferon response
Source: PLoS Pathog. 2026 Feb 17;22(2):e1013987. doi: 10.1371/journal.ppat.1013987 (PMC12923140; doi:10.1371/journal.ppat.1013987)
Supplement: S1 Fig — Intracellular (A) and extracellular (B) viral RNA levels at the indicated time points post-infection were quantified by RT-qPCR. Data are presented as mean ± SD from three independent experiments (n = 3). Statistical analysis was performed using two-way ANOVA; no significant differences were found between the ABT-263-treated and control groups at any time point. (DOCX) [file ppat.1013987.s001.docx]

**
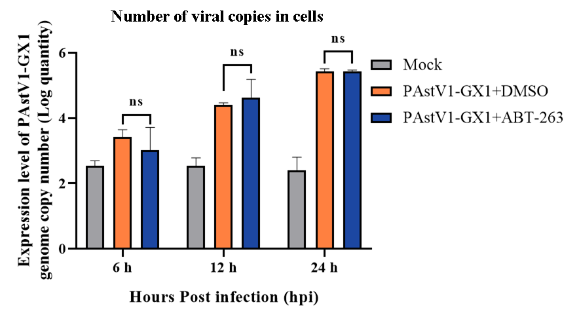

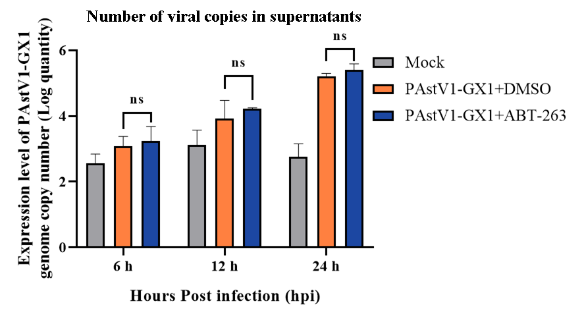
**

B

A

**S1 Fig.**  PK-15 cells were infected with PAstV1-GX1 (MOI = 0.1) and simultaneously treated with or without 5 μM ABT-263. Intracellular (A) and extracellular (B) viral RNA levels at the indicated time points post-infection were quantified by RT-qPCR. Data are presented as mean ± SD from three independent experiments (n=3). Statistical analysis was performed using two-way ANOVA; no significant differences were found between the ABT-263-treated and control groups at any time point.
