# Supplementary material for: The 3C-like serine protease activity of porcine astrovirus nsP1a/3 mediates mitochondrial apoptosis and MAVS cleavage to facilitate viral replication and antagonize type I interferon response
Source: PLoS Pathog. 2026 Feb 17;22(2):e1013987. doi: 10.1371/journal.ppat.1013987 (PMC12923140; doi:10.1371/journal.ppat.1013987)
Supplement: S2 Fig — Cell lysates were subjected to co-immunoprecipitation (co-IP) with anti-Flag or anti-HA beads. The precipitated proteins, along with whole-cell lysates (WCL), were analyzed by Western blot using anti-HA and anti-Flag antibodies. β-Actin was used as a loading control. (DOCX) [file ppat.1013987.s002.docx]

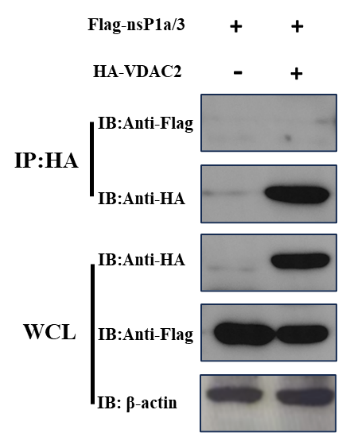

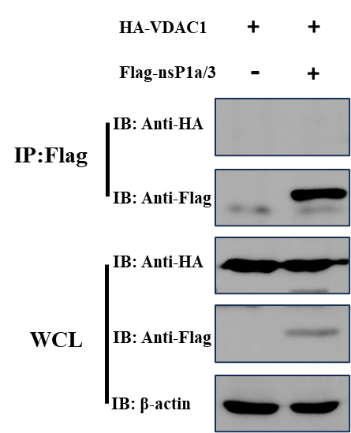

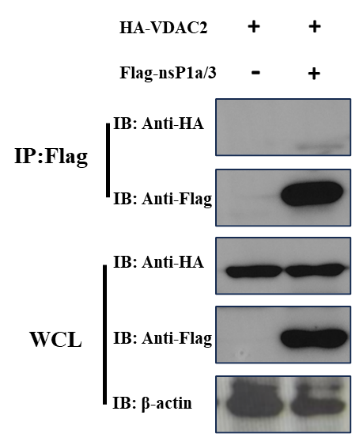


C

A


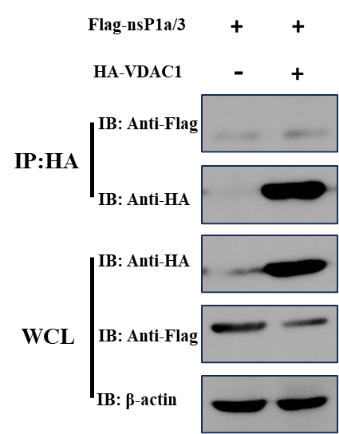


D

B

**S2 Fig.**  HEK-293T cells were co-transfected with plasmids for pCAGGS-Flag-nsP1a/3 and either pCAGGS-HA-VDAC1 (A, B) or pCAGGS-HA-VDAC2 (C, D) for 24 hours. Cell lysates were subjected to co-immunoprecipitation (co-IP) with anti-Flag or anti-HA beads. The precipitated proteins, along with whole-cell lysates (WCL), were analyzed by Western blot using anti-HA and anti-Flag antibodies. β-Actin was used as a loading control.
