# Supplementary material for: The 3C-like serine protease activity of porcine astrovirus nsP1a/3 mediates mitochondrial apoptosis and MAVS cleavage to facilitate viral replication and antagonize type I interferon response
Source: PLoS Pathog. 2026 Feb 17;22(2):e1013987. doi: 10.1371/journal.ppat.1013987 (PMC12923140; doi:10.1371/journal.ppat.1013987)
Supplement: S3 Fig — The 3C-like serine protease region located in the nsP1a/3 is highlighted. (B) Docking complex model of nsP1a/3-MAVS constructed using the AlphaFold 3 engine and PLIP docking algorithm. (DOCX) [file ppat.1013987.s003.docx]

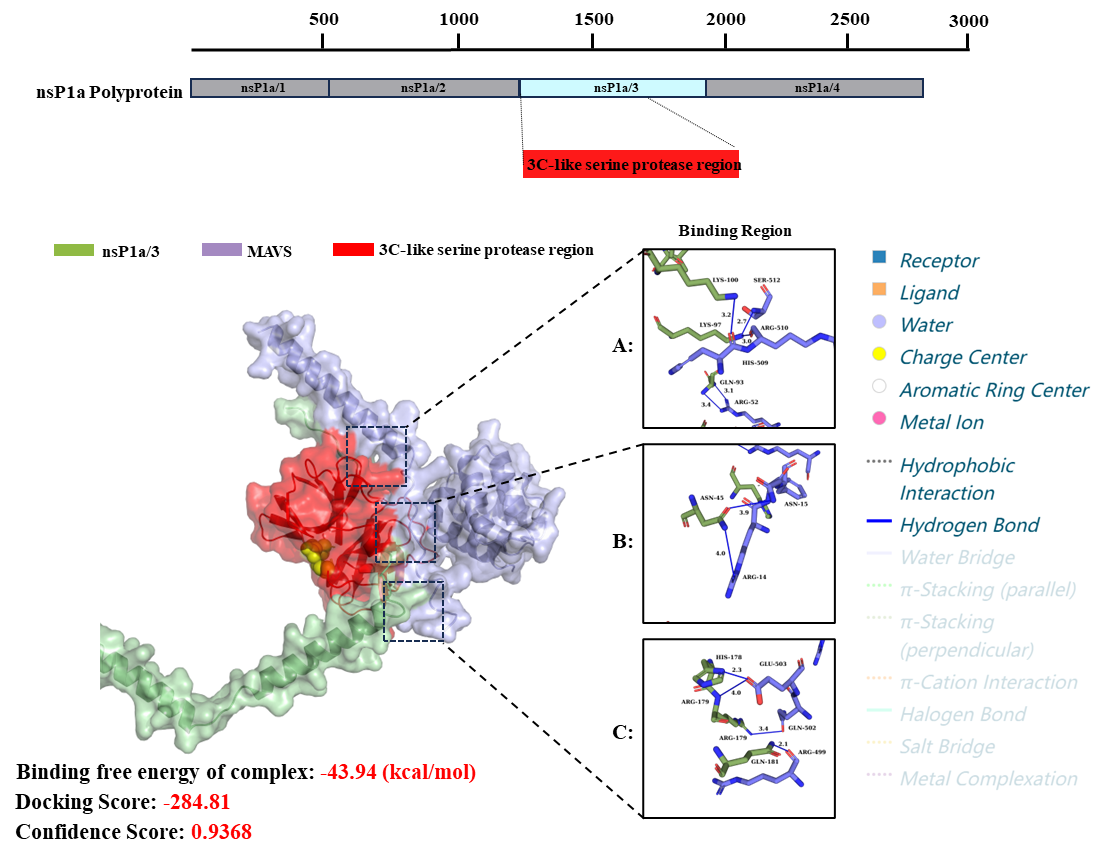


B

A

**S3** **Fig.** (A) Schematic representation of the locations of relevant structural regions within the PAstV nsP1a polyprotein. The 3C-like serine protease region located in the nsP1a/3 is highlighted. (B) Docking complex model of nsP1a/3-MAVS constructed using the AlphaFold 3 engine and PLIP docking algorithm.
