# Supplementary material for: The 3C-like serine protease activity of porcine astrovirus nsP1a/3 mediates mitochondrial apoptosis and MAVS cleavage to facilitate viral replication and antagonize type I interferon response
Source: PLoS Pathog. 2026 Feb 17;22(2):e1013987. doi: 10.1371/journal.ppat.1013987 (PMC12923140; doi:10.1371/journal.ppat.1013987)
Supplement: S4 Fig — Cell lysates were subjected to co-immunoprecipitation (co-IP) using anti-Flag or anti-HA magnetic beads to assess the interaction. Immunoprecipitates and whole-cell lysates (WCL, input) were then analyzed by western blotting with anti-HA and anti-Flag antibodies, respectively. β-actin served as a loading control. (DOCX) [file ppat.1013987.s004.docx]

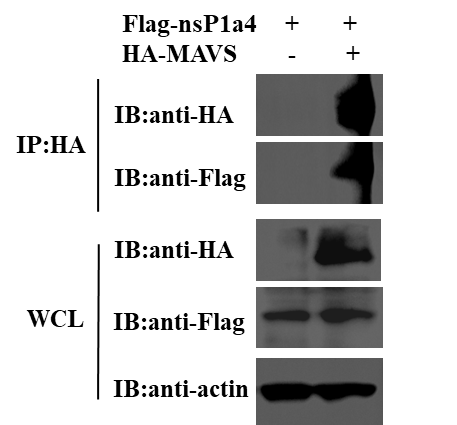

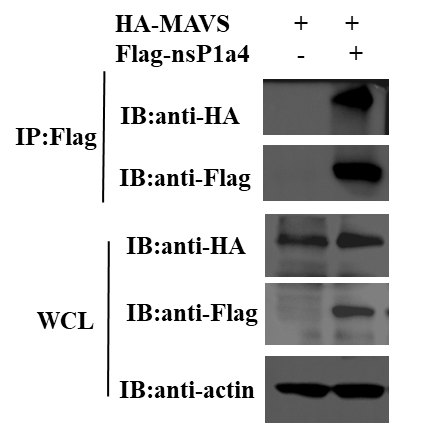
 **S4 Fig.** HEK-293T cells were co-transfected with plasmids expressing Flag-tagged nsP1a/4 and HA-tagged MAVS for 24 h. Cell lysates were subjected to co-immunoprecipitation (co-IP) using anti-Flag or anti-HA magnetic beads to assess the interaction. Immunoprecipitates and whole-cell lysates (WCL, input) were then analyzed by western blotting with anti-HA and anti-Flag antibodies, respectively. β-actin served as a loading control.
