# Supplementary material for: The 3C-like serine protease activity of porcine astrovirus nsP1a/3 mediates mitochondrial apoptosis and MAVS cleavage to facilitate viral replication and antagonize type I interferon response
Source: PLoS Pathog. 2026 Feb 17;22(2):e1013987. doi: 10.1371/journal.ppat.1013987 (PMC12923140; doi:10.1371/journal.ppat.1013987)
Supplement: S5 Fig — (DOCX) [file ppat.1013987.s005.docx]

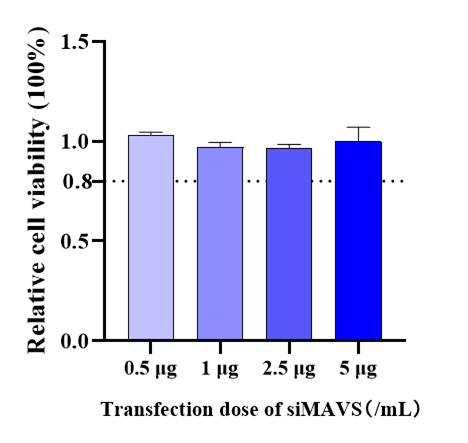


**S5 Fig.** Cell viability assessed by CCK-8 assay in PK-15 cells transfected with graded doses (0.5, 1.0, 2.5, and 5 μg) of siMAVS at 24 hours post-transfection.
