# Supplementary material for: The 3C-like serine protease activity of porcine astrovirus nsP1a/3 mediates mitochondrial apoptosis and MAVS cleavage to facilitate viral replication and antagonize type I interferon response
Source: PLoS Pathog. 2026 Feb 17;22(2):e1013987. doi: 10.1371/journal.ppat.1013987 (PMC12923140; doi:10.1371/journal.ppat.1013987)
Supplement: S6 Fig — Data are expressed as mean ± SD from three independent experiments (n = 3). Statistical significance was assessed by two-way ANOVA. Significant differences compared to the siNC group are denoted as *p < 0.05 and **p < 0.01. (DOCX) [file ppat.1013987.s006.docx]

**
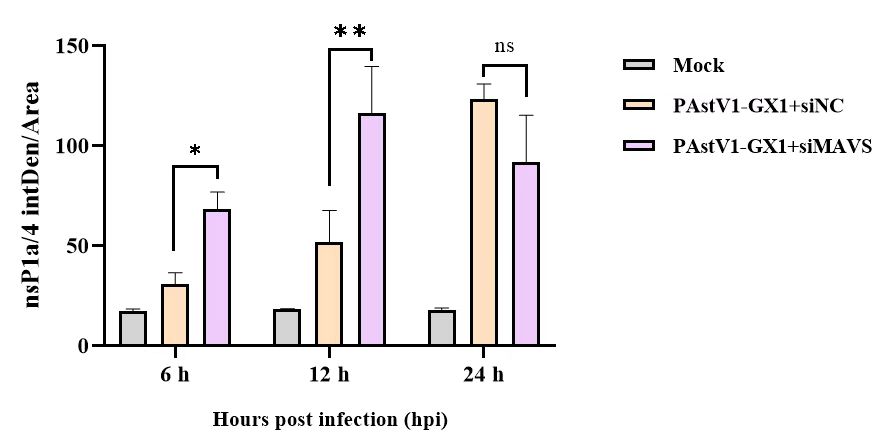
**

**S6 Fig.** Quantification of fluorescence intensity across different treatment groups was performed using ImageJ software. Data are expressed as mean ± SD from three independent experiments (n = 3). Statistical significance was assessed by two-way ANOVA. Significant differences compared to the siNC group are denoted as *p < 0.05 and **p < 0.01.
