# Supplementary material for: The 3C-like serine protease activity of porcine astrovirus nsP1a/3 mediates mitochondrial apoptosis and MAVS cleavage to facilitate viral replication and antagonize type I interferon response
Source: PLoS Pathog. 2026 Feb 17;22(2):e1013987. doi: 10.1371/journal.ppat.1013987 (PMC12923140; doi:10.1371/journal.ppat.1013987)
Supplement: S7 Fig — Cell lysates were collected at 24 h post-transfection and analyzed by Western blot using antibodies against MAVS, Flag, and β-actin. (DOCX) [file ppat.1013987.s007.docx]

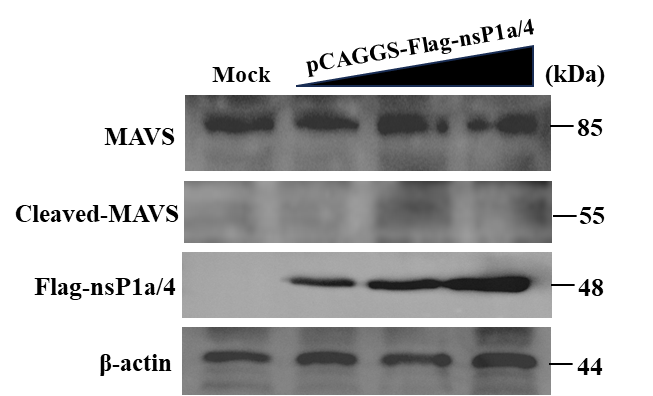


**S7 Fig.**  PK-15 cells were transfected with increasing amounts of the pCAGGS-Flag-nsp1a/4 plasmid, using untransfected cells as negative controls (Mock). Cell lysates were collected at 24 h post-transfection and analyzed by Western blot using antibodies against MAVS, Flag, and β-actin.
