# Supplementary material for: The 3C-like serine protease activity of porcine astrovirus nsP1a/3 mediates mitochondrial apoptosis and MAVS cleavage to facilitate viral replication and antagonize type I interferon response
Source: PLoS Pathog. 2026 Feb 17;22(2):e1013987. doi: 10.1371/journal.ppat.1013987 (PMC12923140; doi:10.1371/journal.ppat.1013987)
Supplement: S8 Fig — Mock represents the uninfected control. Data are expressed as mean ± SD from three independent experiments (n = 3). Statistical significance was assessed by unpaired t‑test. Significant differences compared to the mock group are denoted as ****p < 0.0001. (DOCX) [file ppat.1013987.s008.docx]

 **S8 Fig.** The concentration of porcine IFN‑β in supernatants from SeV‑infected PK‑15 cells was determined using a porcine IFN‑β‑specific ELISA. Mock represents the uninfected control. Data are expressed as mean ± SD from three independent experiments (n = 3). Statistical significance was assessed by unpaired t‑test. Significant differences compared to the mock group are denoted as ****p < 0.0001.
