# Supplementary material for: The 3C-like serine protease activity of porcine astrovirus nsP1a/3 mediates mitochondrial apoptosis and MAVS cleavage to facilitate viral replication and antagonize type I interferon response
Source: PLoS Pathog. 2026 Feb 17;22(2):e1013987. doi: 10.1371/journal.ppat.1013987 (PMC12923140; doi:10.1371/journal.ppat.1013987)
Supplement: S9 Fig — The catalytic triad residues are highlighted in dark color. (DOCX) [file ppat.1013987.s009.docx]

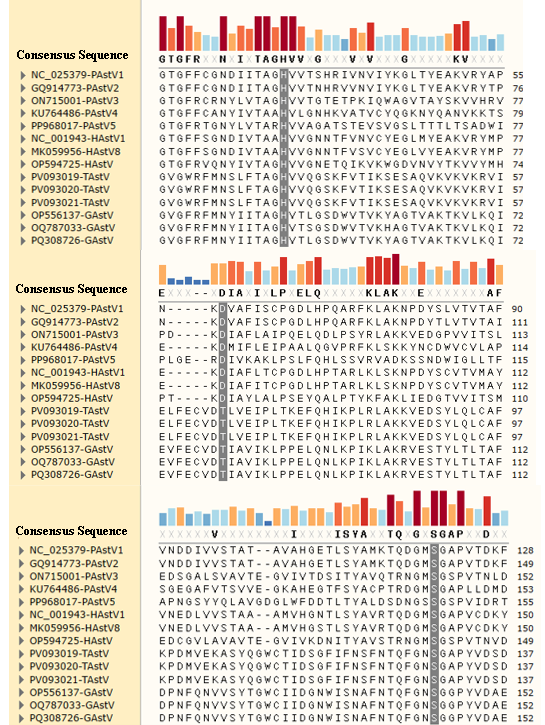


**S9 Fig.** Alignment of the catalytic triad residues in the 3C-like serine protease across different astrovirus species. The catalytic triad residues are highlighted in dark color.
