# Supplementary material for: The 3C-like serine protease activity of porcine astrovirus nsP1a/3 mediates mitochondrial apoptosis and MAVS cleavage to facilitate viral replication and antagonize type I interferon response
Source: PLoS Pathog. 2026 Feb 17;22(2):e1013987. doi: 10.1371/journal.ppat.1013987 (PMC12923140; doi:10.1371/journal.ppat.1013987)
Supplement: S10 Fig — The full-length mRNA sequences of porcine MAVS (GenBank accession no. MK302496.1) and human MAVS (GenBank accession no. KC415005.1) were downloaded from the NCBI database and aligned using the MegAlign software. (DOCX) [file ppat.1013987.s010.docx]

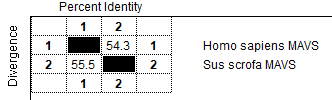


**S10 Fig.**  Full-length amino acid sequence alignment of porcine and human MAVS proteins. The full-length mRNA sequences of porcine MAVS (GenBank accession no. MK302496.1) and human MAVS (GenBank accession no. KC415005.1) were downloaded from the NCBI database and aligned using the MegAlign software.
