# Supplementary material for: The 3C-like serine protease activity of porcine astrovirus nsP1a/3 mediates mitochondrial apoptosis and MAVS cleavage to facilitate viral replication and antagonize type I interferon response
Source: PLoS Pathog. 2026 Feb 17;22(2):e1013987. doi: 10.1371/journal.ppat.1013987 (PMC12923140; doi:10.1371/journal.ppat.1013987)
Supplement: S1 Table — (DOCX) [file ppat.1013987.s011.docx]

S1 Table. Analysis of docking interactions between nsP1a/3 and MAVS.

| **Number (No.)** | **Binding sites** | | **Type of**  **intercropping** | **Location in 3C-like serine protease region** |
| --- | --- | --- | --- | --- |
|  | **nsP1a/3** | **MAVS** |  |  |
| 1 | Lys97 | Ser512 | Hydrogen bond | Yes |
| 2 | Lys97 | Arg510 | Hydrogen bond | Yes |
| 3 | Lys100 | His509 | Hydrogen bond | Yes |
| 4 | Gln93 | Arg52 | Hydrogen bond | Yes |
| 5 | Asn45 | Asn15 | Hydrogen bond | Yes |
| 6 | Asn45 | Arg14 | Hydrogen bond | Yes |
| 7 | His178 | Glu503 | Hydrogen bond | Yes |
| 8 | Arg179 | Glu503 | Hydrogen bond | no |
| 9 | Arg179 | Gln502 | Hydrogen bond | no |
| 10 | Gln181 | Arg499 | Hydrogen bond | no |
