# Supplementary material for: The 3C-like serine protease activity of porcine astrovirus nsP1a/3 mediates mitochondrial apoptosis and MAVS cleavage to facilitate viral replication and antagonize type I interferon response
Source: PLoS Pathog. 2026 Feb 17;22(2):e1013987. doi: 10.1371/journal.ppat.1013987 (PMC12923140; doi:10.1371/journal.ppat.1013987)
Supplement: S2 Table — (DOCX) [file ppat.1013987.s012.docx]

S2 Table. The relative quantitative PCR primers used in this study[16]

| Primer | Sequence (5’ to 3’) |
| --- | --- |
| po-IFN-β-F | AGTGCATCCTCCAAATCGCT |
| po-IFN-β-R | GCTCATGGAAAGAGCTGTGGT |
| po-ISG15-F | CGCAGCAGCCCCTATGAG |
| po-ISG15-R | GACAGCCAGAACTGGTCTGCTT |
| po-ISG56-F | AAATGAATGAAGCCCTGGAGTATT |
| po-ISG56-R | AGGGATCAAGTCCCACAGATTTT |
| po-β-actin-F | GTGATCTCCTTCTGCATCCTGTC |
| po-β-actin-R | GCAAGAACTCACAGGACAGGAA |
